# Redox Regulation, Rather than Stress-Induced Phosphorylation, of a Hog1 Mitogen-Activated Protein Kinase Modulates Its Nitrosative-Stress-Specific Outputs

**DOI:** 10.1128/mBio.02229-17

**Published:** 2018-03-27

**Authors:** Carmen Herrero-de-Dios, Alison M. Day, Anna T. Tillmann, Stavroula L. Kastora, David Stead, Paula S. Salgado, Janet Quinn, Alistair J. P. Brown

**Affiliations:** a Aberdeen Fungal Group, MRC Centre for Medical Mycology, University of Aberdeen, Institute of Medical Sciences, Aberdeen, United Kingdom; b Institute for Cell and Molecular Biosciences, Newcastle University, Newcastle upon Tyne, United Kingdom; c Aberdeen Proteomics, University of Aberdeen, Rowett Institute, Aberdeen, United Kingdom; University of Texas Health Science Center

**Keywords:** *Candida albicans*, MAP kinase signaling, nitrosative stress

## Abstract

In all eukaryotic kingdoms, mitogen-activated protein kinases (MAPKs) play critical roles in cellular responses to environmental cues. These MAPKs are activated by phosphorylation at highly conserved threonine and tyrosine residues in response to specific inputs, leading to their accumulation in the nucleus and the activation of their downstream targets. A specific MAP kinase can regulate different downstream targets depending on the nature of the input signal, thereby raising a key question: what defines the stress-specific outputs of MAP kinases? We find that the Hog1 MAPK contributes to nitrosative-stress resistance in *Candida albicans* even though it displays minimal stress-induced phosphorylation under these conditions. We show that Hog1 becomes oxidized in response to nitrosative stress, accumulates in the nucleus, and regulates the nitrosative stress-induced transcriptome. Mutation of specific cysteine residues revealed that C156 and C161 function together to promote stress resistance, Hog1-mediated nitrosative-stress-induced gene expression, resistance to phagocytic killing, and *C. albicans* virulence. We propose that the oxidation of Hog1, rather than its phosphorylation, contributes to the nitrosative-stress-specific responses of this MAP kinase.

## IMPORTANCE

Mitogen-activated protein kinases play key roles in the responses of eukaryotic cells to extracellular signals and are critical for environmental-stress resistance. The widely accepted paradigm is that MAP kinases are activated by phosphorylation, which then triggers their nuclear accumulation and the activation of target proteins and genes that promote cellular adaptation. Our data suggest that alternative forms of posttranslational modification can modulate MAP kinase functionality in *Candida albicans*. We demonstrate that Hog1 is not significantly phosphorylated in response to nitrosative stress, yet it displays nuclear accumulation and contributes to the global transcriptional response to this stress, as well as promoting nitrosative-stress resistance. Instead, nitrosative stress triggers changes in the redox status of Hog1. We also show that specific Hog1 cysteine residues influence its activation of stress genes. Therefore, alternative posttranslational modifications appear to regulate the stress-specific outputs of MAP kinases.

## INTRODUCTION

To survive, all organisms must respond to environmental change. To achieve this, they activate specific signaling pathways that trigger adaptation to the external challenges, many of which are perceived as an environmental stress. Mitogen-activated protein kinase (MAPK) modules play central roles in many environmental responses in animals, plants, fungi, and protists (1–5).

The initial description of the Hog1 MAP kinase, which is critical for osmo-adaptation in *Saccharomyces cerevisiae* (1), was followed by the discovery of its mammalian homologues p38 and JNK (c-Jun N-terminal kinase) (2, 3). Since then, the molecular and system analysis of fungal MAP kinase modules has contributed greatly to the current understanding of these modules and to stress-signaling networks in general (see, e.g., references 6 to 9). The generally accepted paradigm is that signal perception leads to the activation of a MAP kinase kinase kinase (MAPKKK), which phosphorylates and activates its MAP kinase kinase (MAPKK), which in turn phosphorylates and activates the MAPK and promotes its nuclear accumulation. This holds for the pathogenic yeast *Candida albicans*, where the Pbs2 MAPKK phosphorylates the Hog1 MAPK at the threonine and tyrosine residues in its highly conserved TGY motif in response to a variety of environmental stresses that include osmotic, oxidative, and heavy metal stresses (10, 11). Hog1 phosphorylation leads to its accumulation in the nucleus, the activation of target genes, and the phosphorylation of target proteins, which together contribute to stress adaptation, stress protection, and virulence (12–15).

The paradigm of MAPK phosphorylation and activation is now generally accepted to the extent that Hog1 phosphorylation has become a standard and quantifiable proxy for Hog1 activation (see, e.g., reference 7). However, although *C. albicans* Hog1 is phosphorylated in response to diverse stresses, including osmotic, oxidative, and heavy metal stresses (10), it activates different gene targets in response to these stresses (13). For example, Hog1 plays a significant role in the induction of genes encoding cation exporters and glycerol biosynthetic enzymes in response to osmotic stress and drug transporter and sulfur amino acid biosynthetic genes in response to heavy metal stress. However, Hog1 plays a relatively minor role in the induction of many oxidative-stress-responsive genes following exposure to hydrogen peroxide despite being required for resistance to this oxidative stress, suggesting greater emphasis on posttranscriptional regulation by Hog1 under these conditions (13). Therefore, mechanisms in addition to TGY phosphorylation must control the stress-specific outputs of Hog1. Here, we provide evidence for this in the context of nitrosative stress, which contributes to the antifungal activity of the myeloid cells that are central to our innate immune defenses (16). We show that Hog1 displays minimal levels of phosphorylation following exposure to nitrosative stress yet contributes to the resistance of *C. albicans* to this stress. We also demonstrate that Hog1 becomes oxidized in response to nitrosative stress, that this MAP kinase drives different transcriptional outputs in response to nitrosative and oxidative stresses, and that specific cysteine residues in Hog1 affect these nitrosative- and oxidative-stress outputs. Therefore, modifications at non-TGY residues influence the stress-specific outputs of a MAPK *in vivo*.

## RESULTS

### Hog1 contributes to nitrosative-stress resistance.

*C. albicans* exists as a relatively harmless commensal in most healthy individuals and thus has evolved to evade or resist attack by our immune defenses. Macrophages and neutrophils exploit a battery of reactive oxidative and nitrosative species (ROS and RNS) in their attempts to kill invading microbes (16). However, compared to nonpathogenic fungi, *C. albicans* displays relatively high levels of resistance to these chemical species (17, 18). The resistance of *C. albicans* to oxidative stress is mediated mainly by AP-1 (Cap1) and Hog1 signaling (10, 19–23), and accordingly, Cap1 and Hog1 promote fungal survival following phagocytic attack (22, 24, 25). The resistance to nitrosative stresses is dependent on the transcription factor Cta4 (26), which drives the transcriptional response to nitrosative stress and the activation of *YHB1*, which encodes a conserved flavohemoglobin, nitric oxide dioxygenase, that detoxifies nitric oxide (NO) (27, 28).

**FIG 1:**
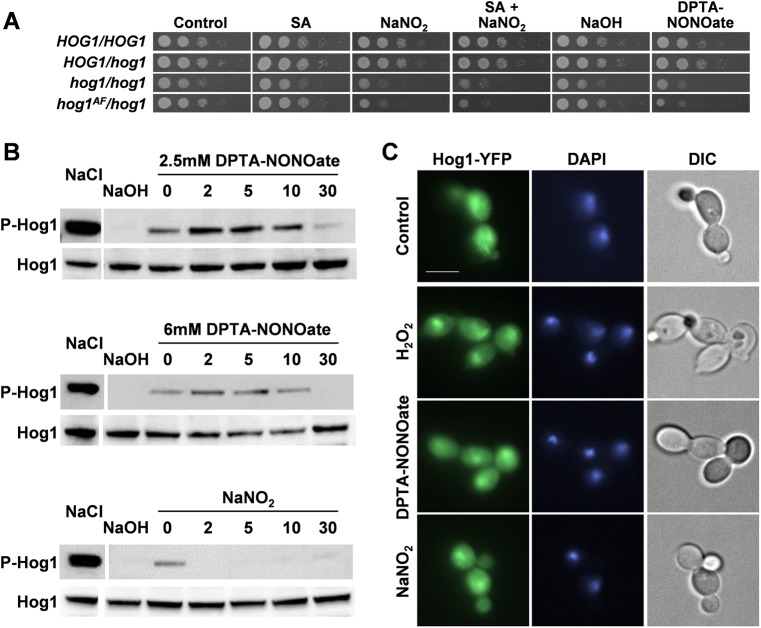
Hog1 is required for nitrosative-stress resistance. (A) To assay stress resistance, the following *C. albicans* strains were spotted in 10-fold serial dilutions on plates containing no stress (control), 25 mM succinic acid (SA), 5 mM NaNO_2_, 25 mM succinic acid plus 5 mM NaNO_2_, 0.01 mM NaOH, or 2.5 mM DPTA-NONOate in 0.01 mM NaOH: RM1000+CIp20 (*HOG1/HOG1*), Ca2226 (*HOG1/hog1*), JC50 (*hog1/hog1*), and JC76 (*hog1*^AF^*/hog1*) (see Table S1 in the supplemental material). All figure panels were derived from the same set of plates. (B) To examine Hog1 phosphorylation following exposure to stress, *C. albicans* RM1000+CIp20 cells were exposed to stress and extracts were prepared and subjected to Western blotting with a phospho-p38 antibody to detect phosphorylated Hog1 (P-Hog1) and with an anti-Hog1 antibody to detect total Hog1 levels (Hog1) after 10 min of exposure to 1 M NaCl and 10 min of exposure to the DPTA-NONOate carrier NaOH at 0.01 mM; then, expression was detected after exposure to 2.5 mM DPTA-NONOate, 6.0 mM DPTA-NONOate, or 5 mM NaNO_2_ at the indicated times (in minutes). All figure panels were derived from the same set of Western blots. (C) Localization of Hog1-YFP in *C. albicans* JC63 cells exposed for 10 min to no stress (control), 5 mM H_2_O_2_, 2.5 mM DPTA-NONOate, or 5 mM NaNO_2_. Nuclei were counterstained with DAPI. Representative images of cells examined by differential interference contrast (DIC) and fluorescence microscopy (Hog1-YFP and DAPI) are shown.

Previously, Hog1 was not thought to contribute significantly to the nitrosative-stress response in *C. albicans*, in part because the inactivation of this MAP kinase does not appear to block the nitrosative-stress-mediated induction of a *YHB1-RLUC* reporter (24). However, we observed that *C. albicans hog1Δ* cells are sensitive to NO and nitrite (Fig. 1A; see also Table S1 in the supplemental material), suggesting that Hog1 contributes to nitrosative-stress resistance. This contribution appears to be dependent on phosphorylation at the TGY motif because mutation of these threonine and tyrosine phosphorylation sites in Hog1 to nonphosphorylatable residues (T174A, Y176F) (15) renders the resulting *hog1*^AF^ cells sensitive to nitrosative stress (Fig. 1A). Yet, compared to the strong phosphorylation observed in response to osmotic stress (1 M NaCl for 10 min), Hog1 displayed minimal levels of phosphorylation following exposure to 5 mM sodium nitrite or to 2.5 or 6 mM dipropylenetriamine (DPTA)-NONOate (Fig. 1B), which represent medium-to-high levels of this NO donor (29). Despite this minimal phosphorylation, Hog1-yellow fluorescent protein (YFP) accumulated in the nucleus in response to a nitrosative stress (Fig. 1C and S1). We conclude that Hog1 accumulates in the nucleus in response to nitrosative stress and contributes to nitrosative-stress resistance but that Hog1 is not strongly phosphorylated in response to this stress.

10.1128/mBio.02229-17.1 FIG S1  Hog1-YFP accumulates in the nucleus following nitrosative stress. Download FIG S1, TIF file, 0.3 MB. Copyright © 2018 Herrero-de-Dios et al. 2018 Herrero-de-Dios et al. This content is distributed under the terms of the Creative Commons Attribution 4.0 International license.

10.1128/mBio.02229-17.6 TABLE S1  *C. albicans* strains and primers. Download TABLE S1, PDF file, 0.1 MB. Copyright © 2018 Herrero-de-Dios et al. 2018 Herrero-de-Dios et al. This content is distributed under the terms of the Creative Commons Attribution 4.0 International license.

### Hog1 contributes to the transcriptional response to nitrosative stress.

**FIG 2:**
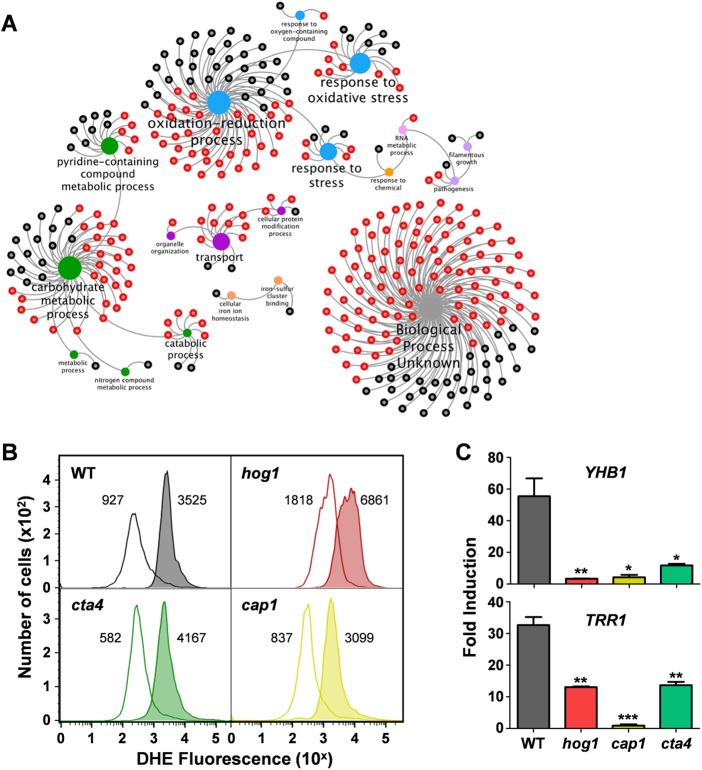
Hog1 contributes to the transcriptional response to nitrosative stress. (A) Transcript profiling (RNA sequencing) was performed on three independent replicates cultures of *C. albicans HOG1* (RM1000+Clp20) (Table S1) and *hog1Δ* cells (JC50) exposed to 0 or 2.5 mM DPTA-NONOate for 10 min. In wild-type cells, 321 transcripts displayed statistically significant (>2-fold) increases in level in response to the nitrosative stress. Of these, 205 transcripts (64%) were considered to be Hog1-dependent (red nodes) because they were not induced ≥2-fold by nitrosative stress in *hog1Δ* cells. The remaining 116 transcripts were considered to be Hog1 independent because they were still induced >2-fold in *hog1Δ* cells. Using Cytoscape, GO term analysis was performed on these gene subsets, and the outputs were displayed as a gene function network in which the red nodes represent Hog1-dependent genes, the dark grey nodes represent Hog1-independent genes, and the central hubs are colored according to the functional category. For example, blue hubs relate to stress, green hubs relate to metabolism, and the light-gray hub is for genes of unknown function. (B) The levels of intracellular ROS were assayed in *C. albicans* strains exposed to 0 or 2.5 mM DPTA-NONOate by performing cytometry on DHE-stained cells. Wild type (WT), gray; *hog1*Δ, red; *cta4*Δ, green; *cap1*Δ, yellow. DHE intensity is presented on a log scale, and the mean fluorescent intensity for each cytometry profile is shown. The data shown are representative of three independent experiments. (C) The fold induction of classical nitrosative-stress (*YHB1*) and oxidative-stress (*TRR1*) transcripts is shown following exposure of the same *C. albicans* strains to 2.5 mM DPTA-NONOate for 30 min. Using qRT-PCR, transcript levels were measured relative to the internal *ACT1* mRNA control and normalized to the level of that transcript in the absence of the stress. Means and standard deviations are shown for three independent replicate experiments. *, *P* < 0.05; **, *P* < 0.01; ***, *P* < 0.001.

Given that this MAP kinase accumulated in the nucleus after exposure to nitrosative stress, we wondered whether Hog1 contributes to the transcriptional response to this stress. To test this, we performed transcript profiling on *C. albicans* wild-type and *hog1Δ* cells exposed to 0 or 2.5 mM DPTA-NONOate by RNA sequencing. In wild-type cells, 321 genes consistently displayed a ≥2-fold increase in expression in response to this stress (Table S2). Hromatka and coworkers (28) described fewer nitrosative-stress-induced genes in their microarray analysis of the transcriptional response to nitrosative stress in *C. albicans*. This is to be expected given (i) the increased depth of coverage and higher dynamic range observed for RNA sequencing studies (30) and (ii) the lower dose that they examined in their study (1.0 versus 2.5 mM DPTA-NONOate). Of the 40 genes that were induced over 3-fold by nitrosative stress in their microarray study (see Table 2 in reference 28, 80% were upregulated in response to nitrosative stress in our study (Table S3). *YHB1* was among the most strongly regulated genes in both studies (Table S3). Furthermore, both studies found that, in addition to *YHB1*, genes involved in oxido-reduction, the oxidative-stress response, and carbohydrate metabolism were induced in response to nitrosative stress (Fig. 2A; Table S3).

10.1128/mBio.02229-17.7 TABLE S2  Nitrosative-stress-regulated genes in *C. albicans* revealed by RNA sequencing. Download TABLE S2, PDF file, 0.1 MB. Copyright © 2018 Herrero-de-Dios et al. 2018 Herrero-de-Dios et al. This content is distributed under the terms of the Creative Commons Attribution 4.0 International license.

10.1128/mBio.02229-17.8 TABLE S3  Comparison of nitrosative-stress-induced genes observed in this study with those identified by Hromatka and coworkers (28). Nitrosative-stress genes in this study (upregulated >2-fold) are compared with nitrosative-stress genes from the work of Hromatka et al. (28) (upregulated >log_2_-fold). Of the 66 genes that were upregulated in the Hromatka et al. study, 35 (53%) were upregulated in this study (highlighted in blue), including *YHB1*. Download TABLE S3, PDF file, 0.1 MB. Copyright © 2018 Herrero-de-Dios et al. 2018 Herrero-de-Dios et al. This content is distributed under the terms of the Creative Commons Attribution 4.0 International license.

We observed that the induction of a significant proportion of the nitrosative-stress-induced genes (64%) was attenuated in *hog1Δ* cells (Fig. 2A; Table S4) indicating that, despite the low levels of Hog1 phosphorylation in response to nitrosative stress (Fig. 1B), this MAP kinase makes a significant contribution to the transcriptional response to nitrosative stress in *C. albicans*. We compared the 207 Hog1-dependent nitrosative-stress genes identified by RNA sequencing in this study with the Hog1-dependent oxidative- and osmotic-stress genes identified in our earlier microarray study (13). Fewer Hog1-dependent stress genes were observed in this microarray study than in our current RNA sequencing study (Table S4), no doubt because of the higher dynamic range of RNA sequencing (30). Nevertheless, only half of the Hog1-dependent oxidative-stress-induced genes and one-fifth of the Hog1-dependent osmotic-stress-induced genes observed on the microarrays also displayed Hog1-dependent induction in response to nitrosative stress (Table S4). Forty-three Hog1-dependent nitrosative-stress genes retained some degree of nitrosative-stress activation in *hog1*Δ cells (Table S5). Of these 43 genes, 19 are induced in response to oxidative stress and 3 are upregulated by osmotic stress (Table S5). This suggests partial overlap between the Hog1 outputs for these different stress conditions.

10.1128/mBio.02229-17.9 TABLE S4  Comparison of Hog1-dependent nitrosative-stress-induced genes with Hog1-dependent oxidative-stress- and osmotic-stress-induced genes identified by Enjalbert and coworkers (13). Hog1 dependence was defined as a >2-fold decrease in nitrosative-stress gene induction following Hog1 inactivation (i.e., in *hog1* cells compared to wild-type cells). Download TABLE S4, PDF file, 0.1 MB. Copyright © 2018 Herrero-de-Dios et al. 2018 Herrero-de-Dios et al. This content is distributed under the terms of the Creative Commons Attribution 4.0 International license.

10.1128/mBio.02229-17.10 TABLE S5  Oxidative- and osmotic-stress responses of Hog1-dependent genes that retain inducibility in response to nitrosative stress. Download TABLE S5, PDF file, 0.1 MB. Copyright © 2018 Herrero-de-Dios et al. 2018 Herrero-de-Dios et al. This content is distributed under the terms of the Creative Commons Attribution 4.0 International license.

As stated, genes involved in oxido-reduction and the oxidative-stress response were induced in response to nitrosative stress (Fig. 2A). This was to be expected because NO inhibits mitochondrial oxidative phosphorylation, thereby generating superoxide, peroxynitrite, and hydrogen peroxide (31, 32). Consistent with this, we also observed that exposure to nitrosative stress led to an increase in the intracellular levels of ROS in the wild type. This was also the case in *hog1*Δ, *cta4*Δ, and *cap1*Δ cells (Fig. 2B), which lack key regulators of the oxidative- and nitrosative-stress responses (10, 13, 19, 21, 26). Interestingly, the loss of Cta4 led to a 7.2-fold increase in ROS after the nitrosative stress, whereas the other strains displayed a 3.7-fold increase, largely because basal ROS levels were lower in *cta4*Δ cells (Fig. 2B). Most importantly, the data suggest that the nitrosative-stress-mediated induction of oxidative-stress genes might be triggered by the elevation of ROS in these strains. In addition, nitrosative stress causes molecular damage, such as protein *S-*nitrosylation. Accordingly, we observed the induction of genes involved in glutathione synthesis and recycling (*GCS1*, *GTT1*, *GST2*) and the glutaredoxin and thioredoxin systems (*GRX2*, *GPX3*, *TRR1*) in response to nitrosative stress (Table S2). Therefore, we tested the relative contributions of Hog1, Cta4, and Cap1 to the induction of classic nitrosative (*YHB1*)- and oxidative (*TRR1*)-stress genes. Interestingly, all three regulators contributed significantly to the nitrosative-stress-mediated induction of both *YHB1* and *TRR1* (Fig. 2C). This suggests that nitrosative stress generates intracellular ROS, which leads to Cap1 activation, which then contributes to the transcriptional response to the nitrosative stress together with Cta4. We also conclude that Hog1 makes a significant contribution to the activation of some oxidative-stress genes, as well as nitrosative-stress genes, in response to nitrosative stress. This was unexpected because Hog1 is not required for the induction of most oxidative-stress genes in response to oxidative stress (13) (Table S4). These observations are consistent with the idea that Hog1 mediates different outputs in response to nitrosative and oxidative stresses.

### Hog1 is oxidized in response to nitrosative stress.

**FIG 3:**
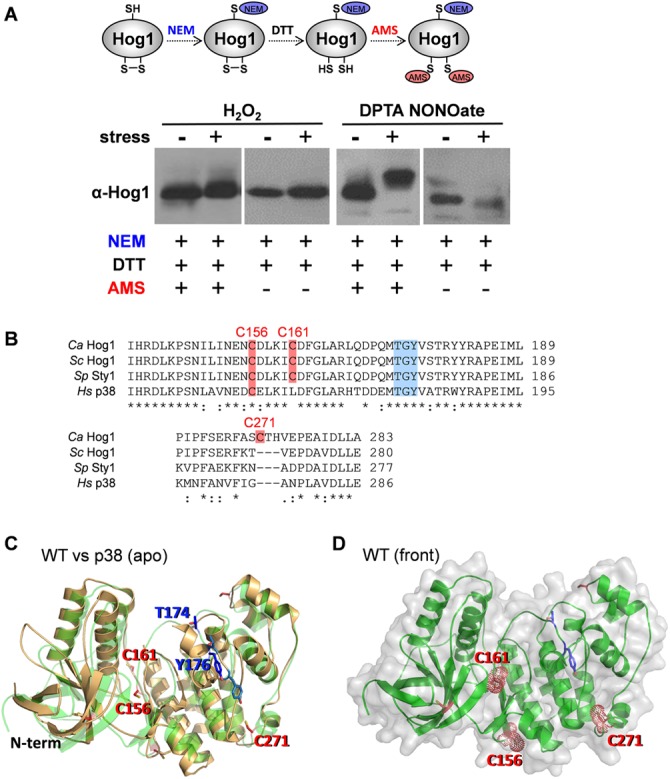
Redox status of Hog1 following nitrosative stress. (A) The impact of oxidative stress (a 10-min exposure to 5 mM H_2_O_2_) and nitrosative stress (a 10-min exposure to 2.5 mM DPTA-NONOate) on the redox status of Hog1 in wild-type *C. albicans* cells (RM1000+Clp20) (Table S1) was analyzed by Western blotting of AMS- and NEM-alkylated extracts with the anti-Hog1 antibody. As shown in the cartoon, proteins were first alkylated with NEM (<0.1 kDa) to block free thiols. Disulfide bonds were then reduced, and the newly exposed thiols were alkylated with AMS (~0.5 kDa). Control extracts that were not alkylated with AMS show that, in unstressed *C. albicans* cells, no disulfide bonds exist in Hog1. The increase in Hog1 mass following DPTA-NONOate treatment indicates the formation of a new disulfide bond(s) in these cells. (B) Comparisons of parts of the amino acid sequences of *C. albicans* (*Ca*) and *S. cerevisiae* (*Sc*) Hog1, *S. pombe* (*Sp*) Sty1, and *Homo sapiens* (Hs) p38, showing the juxtapositions of C156, C161, and C271 (pink) relative to the TGY phosphorylation motif (blue) in evolutionarily conserved stress-activated protein kinases. *, identical residue; :, conserved residue. (C) Comparison of the predicted structural model of *C. albicans* Hog1 (green) against the human p38 (gold) model (PDB accession number 1R39) indicates that their structures are very similar, with the same overall fold. The threonine and tyrosine residues in the TGY motif are highlighted in blue (stick representation), and the relevant cysteine residues are highlighted in red. A slightly less exposed positioning of these residues is predicted in Hog1. (D) Cartoon and surface representation of the predicted structural modeling of *C. albicans* Hog1 shows the positions of the targeted cysteines (red-dot spheres, C156, C161, and C271), relative to the TGY phosphorylation motif (stick representation, blue). N-term, N terminus.

Hog1 displays minimal levels of phosphorylation in response to nitrosative stresses compared with the strong phosphorylation observed with osmotic stress (Fig. 1B), yet Hog1 contributes to the transcriptional response to nitrosative stress (Fig. 2). Might Hog1 be activated by some other means in response to this stress? In mammalian systems, *S*-nitrosylation or oxidation of redox-sensitive cysteine residues is emerging as an important signaling mechanism following nitrosative and oxidative stresses (33–36), and the mammalian MAP kinases JNK1 and p38 are negatively regulated by *S*-nitrosylation and cysteine oxidation (37–39). Also, oxidative-stress-induced disulfide bond formation between redox-sensitive cysteine residues in the Hog1 orthologue Sty1 positively regulates the transcriptional response to oxidative stress in *Schizosaccharomyces pombe* (40). The oxidation of redox-sensitive thiol groups to the unstable sulfenic form often triggers the formation of the more stable disulfide bond. In addition, the *S*-nitrosylation of cysteine residues is often the precursor of disulfide bond formation (41, 42). Therefore, we probed for nitrosative-stress-induced changes in disulfide bond formation within Hog1 by treating *C. albicans* protein extracts sequentially with *N*-ethylmaleimide (NEM), dithiothreitol (DTT), and 4-acetamido-4′-maleimidylstilbene-2,2′-disulfonic acid (AMS) (23). Both NEM and AMS specifically alkylate sulfhydryl groups on reduced cysteine residues, but while NEM alkylation has a minimal effect on a protein’s mass, AMS alkylation adds 0.6 kDa per modified cysteine (43). First, we treated extracts with NEM to block reduced cysteines (Fig. 3A). Then, these extracts were treated with DTT to reduce any disulfides, and the released cysteine residues were then alkylated with AMS (Fig. 3A). Therefore, AMS-dependent increases in the molecular mass of a protein, as detected by a reduced mobility in SDS-PAGE, indicate the presence of disulfide bonds in that protein.

Western blotting of NEM-DTT-AMS-treated *C. albicans* extracts revealed that, unlike with Sty1 in *S. pombe* (40), oxidative stress does not induce disulfide bond formation in Hog1 (Fig. 3A). This was first observed using 5 mM H_2_O_2_ but was also the case even when *C. albicans* cells were exposed to a high dose of H_2_O_2_ (30 mM) (Fig. 3A). In contrast, such high levels of H_2_O_2_ rapidly induce oxidation of Sty1 in *S. pombe* (40). Interestingly, however, there was a clear induction of disulfide bond formation in Hog1 after *C. albicans* cells were exposed to 2.5 mM DPTA-NONOate. This nitrosative stress caused an AMS-dependent increase in the molecular mass of Hog1 (Fig. 3A), indicating that the stress had led to disulfide bond formation in this MAP kinase. In principle, this disulfide bond formation might represent *S*-nitrosylation, as *S*-nitrosylation at cysteine residues can lead to disulfide bond formation (41, 42).

In *S. pombe* Sty1, the cysteine residues C153 and C158 form a disulfide in response to oxidative stress (40). These residues lie in a highly evolutionarily conserved region of this MAP kinase (Fig. 3B), and therefore, we targeted the corresponding residues in *C. albicans* Hog1 for analysis (C156 and C161). In addition, we performed proteomics to test whether other Hog1 residues might be subject to modification in response to nitrosative stress. Briefly, *C. albicans* cells expressing tandem-affinity purification (TAP)-tagged Hog1 (JC310) (Table S1) were treated with 0 or 2.5 mM DPTA-NONOate, Hog1 was purified from protein extracts by tandem-affinity purification, and the Hog1 tryptic peptides were subjected to mass spectrometry to detect irreversible nitrosylation or oxidation events (see Materials and Methods). C156- and C161-containing tryptic peptides were observed (DLKPSNILINENCDLK; ICDFGLAR), but no irreversible oxidation events, such as cysteine hyperoxidation, were detected on these peptides. (Reversible oxidation events, such as disulfide bond formation, would not have been detected because the sample preparation included reduction with DTT and *S*-alkylation with iodoacetamide.) However, we did detect hyperoxidation of Hog1 C271 to a sulfonic acid residue (−SOHO_2_) in cells exposed to the nitrosative stress (Fig. S2). Therefore, we also targeted C271 for analysis. Unlike C156 and C161, C271 is not conserved in the Hog1 orthologues from *S. cerevisiae*, *S. pombe*, and humans (Fig. 3B).

10.1128/mBio.02229-17.2 FIG S2  Hog1 C271 becomes oxidized following nitrosative stress. Download FIG S2, TIF file, 1 MB. Copyright © 2018 Herrero-de-Dios et al. 2018 Herrero-de-Dios et al. This content is distributed under the terms of the Creative Commons Attribution 4.0 International license.

### C156, C161, and C271 exert differential effects on Hog1 outputs.

Mutated versions of *C. albicans* Hog1 were created by converting the cysteines at C156, C161, and C271 to serines, and these mutations were confirmed by resequencing. We performed structural modeling of *C. albicans* Hog1 using Phyre2 and compared it to the available crystal structure of a closely related protein, human p38, suggesting a conserved overall fold and very similar structures (Fig. 3C). The model revealed the likely juxtapositions of these three cysteine residues and the TGY motif within the Hog1 structure (Fig. 3D). Also, our modeling of the Hog1^C156S^, Hog1^C161S^, and Hog1^C271S^ proteins suggested that the C156S, C161S, and C271S mutations are unlikely to cause major perturbations in the overall structure (Fig. S3).

10.1128/mBio.02229-17.3 FIG S3  The C→S mutations are unlikely to affect Hog1 structure. Download FIG S3, TIF file, 1.3 MB. Copyright © 2018 Herrero-de-Dios et al. 2018 Herrero-de-Dios et al. This content is distributed under the terms of the Creative Commons Attribution 4.0 International license.

**FIG 4:**
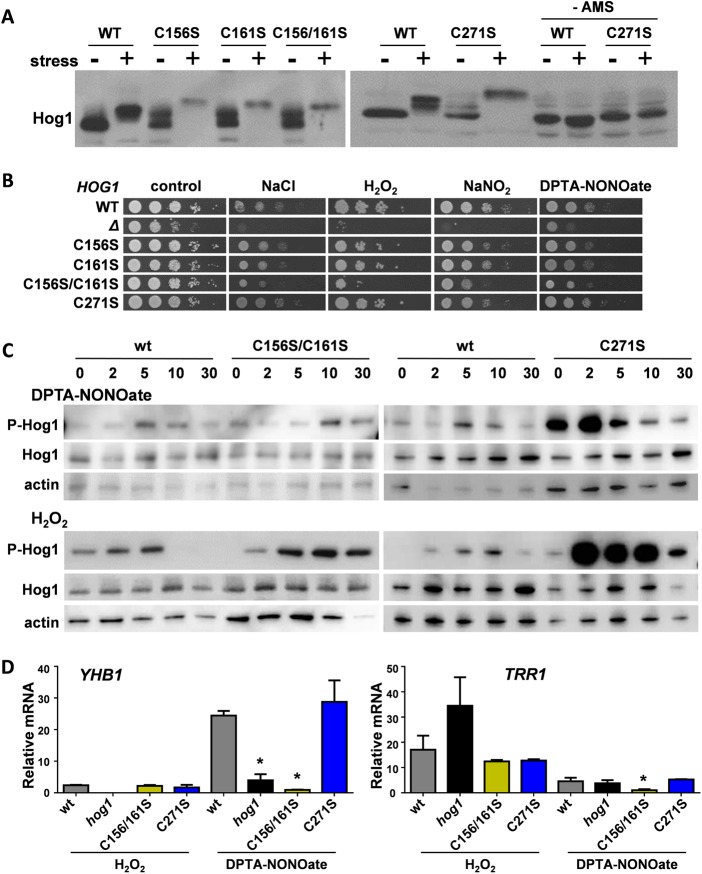
Stress-specific Hog1 outputs are differentially affected by C156, C161, and C271. (A) Impact of C156S, C161S, and C271S mutations upon the redox status of Hog1 following 10 min of exposure to 2.5 mM DPTA-NONOate, as revealed by AMS gels (see the legend to Fig. 3A). The differences in Hog1 masses between wild-type cells and *HOG1*^C→S^ mutants reflect changes in disulfide bond formation in Hog1 in the absence and presence of stress. WT, Ca2226; C156S, Ca2222; C161S, Ca2224; C156/161S, Ca2225; C271S, Ca2216 (Table S1). For the C156/161S columns, the wild-type controls were from the same blot as that showing the lanes with (+) and without (−) DPTA-NONOate and treatment with NEM, DTT, and AMS in Fig. 3A. (B) Impact of C156S, C161S, and C271S mutations upon the nitrosative-, oxidative-, and osmotic-stress sensitivity of *C. albicans*. Strains were spotted in 10-fold serial dilutions onto plates containing no stress (control), 1 M NaCl, 5 mM H_2_O_2_, 5 mM NaNO_2_, or 2.5 mM DPTA-NONOate. All figure panels for the wild type, null mutant, and C156S and C161S single and double mutants were derived from the same set of plates. The C271 mutant, which displayed no obvious stress phenotype, was compared to the wild-type control in a separate experiment. (C) Impact of C156S, C161S, and C271S mutations upon the phosphorylation dynamics of Hog1 phosphorylation following exposure of cells to 2.5 mM DPTA-NONOate or 5 mM H_2_O_2_. Western blotting was performed on cell extracts prepared at the times indicated (in minutes). Phosphorylated Hog1 (P-Hog1) was detected by probing the blots with phospho-p38 antibody, and for loading controls, the blots were reprobed for total Hog1 and actin. The results are indicative of three independent replicate experiments, and the data for the individual *HOG1*^C156S^ and *HOG1*^C161S^ mutants are shown in Fig. S4. (D) Impact of C156S, C161S, and C271S mutations upon the Hog1-mediated induction of nitrosative (*YHB1*)- and oxidative (*TRR1*)-stress genes in response to 5 mM H_2_O_2_ or 2.5 mM DPTA-NONOate for 10 min. The strains are as described for panel A. The fold induction of the *YHB1* and *TRR1* transcripts was measured by qRT-PCR, relative to the internal *ACT1* mRNA control, and by normalizing to the levels of these transcripts in the absence of the stress. Means and standard deviations are shown for three independent replicate experiments. *, *P* < 0.05.

We sequentially treated samples with NEM, DTT, and AMS and then subjected them to gel electrophoresis to test whether these mutations affect the redox state of Hog1 (Fig. 4A). This revealed that the single C156S and C161S mutations promoted disulfide bond formation in Hog1 even in the absence of stress, as a significant fraction of Hog1 displayed an AMS-dependent decrease in Hog1 mobility following NEM-DTT-AMS treatment. However, neither mutation blocked the nitrosative-stress-induced changes in Hog1’s redox state (Fig. 4A). Also, neither the C156S nor the C161S mutation affected the stress sensitivity of *C. albicans* when the phenotypes of the single *HOG1*^C156S^ and *HOG1*^C161S^ mutants were compared with those of wild-type and *hog1* control strains following osmotic (1 M NaCl), oxidative (5 mM H_2_O_2_), or nitrosative (5 mM NaNO_2_ and 6 mM DPTA-NONOate) stress (Fig. 4B). Moreover, these mutations did not affect the phosphorylation dynamics of Hog1 in response to nitrosative or oxidative stress (Fig. S4). Therefore, individually, these mutations do not appear to affect Hog1 functionality. The C156S C161S double mutation resulted in changes in Hog1’s redox state similar to those produced by the corresponding single mutations (Fig. 4A) and exerted only minor effects upon Hog1 phosphorylation dynamics, with Hog1^C156S C161S^ displaying protracted phosphorylation in response to oxidative stress (Fig. 4C). However, the *HOG1*^C156S C161S^ double mutation attenuated the resistance of *C. albicans* to osmotic, oxidative, and nitrosative stresses, albeit not as strongly as a *hog1* null mutation (Fig. 4A). It has been reported that the catalytic activity of the p38 MAP kinase remains unaffected by a C162S mutation but that this mutation influences the structure of its docking site for substrates and activators (64). Therefore, it is conceivable that, while single C156S and C161S mutations do not significantly affect Hog1 function, the double mutation subtly alters the structure of Hog1 and hence its functionality, thereby attenuating the stress resistance of *C. albicans*.

10.1128/mBio.02229-17.4 FIG S4  Impact of C156S and C161S on Hog1 phosphorylation. Download FIG S4, TIF file, 0.5 MB. Copyright © 2018 Herrero-de-Dios et al. 2018 Herrero-de-Dios et al. This content is distributed under the terms of the Creative Commons Attribution 4.0 International license.

The C271S mutation did not block disulfide bond formation in Hog1 in the absence of stress (Fig. 4A) but did exert a dramatic effect on Hog1 phosphorylation (Fig. 4C). Compared to the wild-type control, Hog1^C271S^ displayed increased phosphorylation levels after nitrosative stress and strong phosphorylation in response to oxidative stress (Fig. 4C). These *HOG1*^C271S^ cells also displayed wild-type levels of resistance to nitrosative and oxidative stresses (Fig. 4B). Clearly, the C156S C161S double and C271 single mutations impose different effects upon Hog1 functionality.

To test this further, we examined the impact of these mutations on Hog1 target genes by quantitative reverse transcription-PCR (qRT-PCR). Hog1 contributes to the activation of the nitrosative-stress gene *YHB1* following exposure to a nitrosative stress (Fig. 2C; Table S4). As expected (27, 28), *YHB1* was strongly induced by DPTA-NONOate in wild-type cells (Fig. 4D). This induction was blocked in the *HOG1*^C156S C161S^ mutant but not in *HOG1*^C271S^ cells (Fig. 4D). *YHB1* displayed limited induction in response to H_2_O_2_. The activation of *YHB1* in response to oxidative stress was not affected significantly by the *HOG1*^C156S C161S^ and *HOG1*^C271S^ mutations (Fig. 4D).

We also tested the effects of the *HOG1*^C→S^ mutants on the induction of the thioredoxin reductase (*TRR1*) gene. As expected (13, 14), the expression of this classic oxidative-stress gene was induced by H_2_O_2_ (Fig. 4D). *TRR1* activation in response to oxidative stress was not affected in *HOG1*^C156S C161S^ or *HOG1*^C271S^ cells (Fig. 4D). *TRR1* was also induced by DPTA-NONOate in wild-type cells (Fig. 2C and 4D). Interestingly, this induction was blocked in the *HOG1*^C156S C161S^ mutant but not in *HOG1*^C271S^ cells (Fig. 4D). Taken together, the data indicate that the *HOG1*^C271S^ mutation does not affect *TRR1* or *YHB1* activation in response to nitrosative or oxidative stress. In contrast, the *HOG1*^C156S C161S^ mutations block the induction of both genes specifically in response to nitrosative stress, but not oxidative stress.

We conclude that cysteine residues C156, C161, and C271 differentially affect Hog1 functionality. Although none of these cysteine residues contribute to nitrosative-stress-induced oxidation, the C271S mutation triggers aberrantly strong Hog1 phosphorylation in response to oxidative and nitrosative stresses. In addition, although the single C156S and C161S mutations exert no obvious effects upon Hog1 functionality, the double mutations compromise Hog1 function, attenuating stress resistance and inhibiting gene induction specifically in response to nitrosative stress.

### Hog1 C156S, C161S, and C271S mutations differentially affect *C. albicans* virulence.

**FIG 5:**
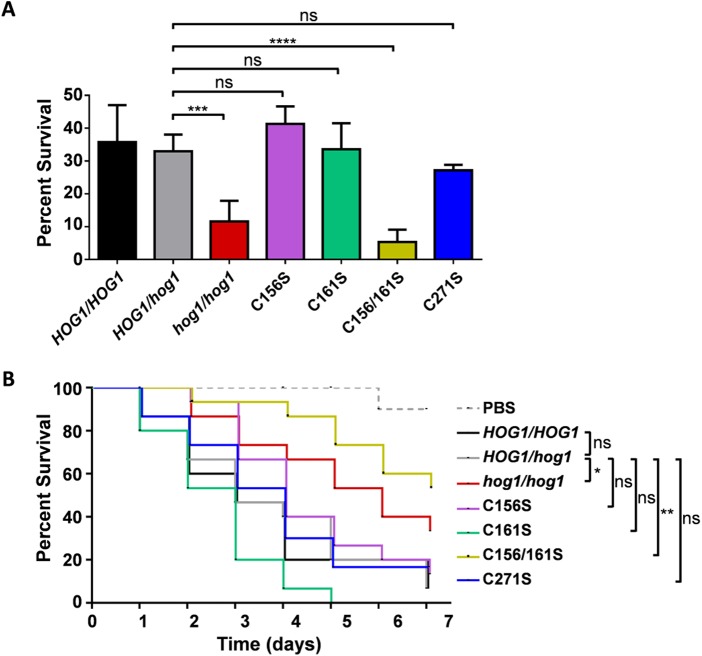
Hog1 C156S, C161S, and C271S mutations differentially impact the sensitivity to phagocytic killing and virulence of *C. albicans*. (A) The sensitivity of *C. albicans* to phagocytic killing was assayed by counting fungal CFU after 2 h of exposure to human PMNs (1:10 ratio of yeast cells to phagocytes). PBS, phosphate-buffered saline control; *HOG1/HOG1*, RM1000+CIp20; *HOG1/hog1*, Ca2226; *hog1/hog1*, JC50; C156S, Ca2222; C161S, Ca2224; C156/161S, Ca2225; C271S, Ca2216 (Table S1). Means and standard deviations from three independent replicate experiments are shown. ns, not significant; ***, *P* < 0.001; ****, *P* < 0.0001. (B) Levels of virulence of the same *C. albicans* strains were compared in the *Galleria* model of systemic infection (20 larvae per fungal strain). The data were analyzed statistically using the log-rank (Mantel-Cox) test. ns, not significant; *, *P* < 0.05; **, *P* < 0.01.

Myeloid cells use combinations of ROS and RNS in their attempts to kill fungal cells and prevent infection (16). *C. albicans* attempts to resist this killing by mounting robust oxidative- and nitrosative-stress responses (44), and Hog1 contributes to this resistance to phagocytic killing (22, 24, 25). Therefore, we tested the ability of the *HOG1*^C→S^ mutants to survive exposure to human polymorphonuclear granulocytes (a 1:10 ratio of fungal cells to polymorphonuclear granulocytes [PMNs] for 2 h). The single *HOG1*^C156S^ and *HOG1*^C161S^ mutants did not attenuate survival, but the *HOG1*^C156S C161S^ double mutant was as sensitive as the *hog1Δ* mutant to phagocytic killing (Fig. 5A). In contrast, *HOG1*^C271S^ cells did not display a significant decrease in survival (Fig. 5A). Taken together, the data suggest that wild-type levels of stress resistance are required for resistance to phagocytic killing.

We then tested the impact of the Hog1^C→S^ mutations upon the virulence of *C. albicans* during systemic infection. We used the invertebrate wax moth (*Galleria mellonella*) model, which is a well-established proxy for systemic infection of the mammalian host (45, 46) and for the impact of Hog1 on systemic infection (47). As expected (12, 15), the inactivation of Hog1 significantly attenuated the virulence of *C. albicans* (Fig. 5B, compare the *HOG1* and *hog1* cells), and the heterozygous null mutant displayed an intermediate level of virulence (Fig. 5B, *HOG1/hog1*Δ cells). Compared to this *HOG1/hog1Δ* control, *HOG1*^C156S^, *HOG1*^C161S^, and *HOG1*^C271S^ cells did not display a significant reduction in virulence. In contrast, the virulence of *HOG1*^C156S C161S^ cells was significantly reduced. These observations were consistent with those from the phagocytic killing assays (above), reinforcing the view that Hog1 functionality and stress resistance are important for the virulence of *C. albicans*.

## DISCUSSION

Our observations show clearly that the Hog1 MAP kinase contributes to nitrosative-stress adaptation and resistance in the major fungal pathogen of humans, *C. albicans*. The inactivation of Hog1 confers sensitivity to RNS (Fig. 1A) and compromises the global transcriptional response to this type of stress (Fig. 2). This extends the breadth of roles already ascribed to this MAP kinase, which include contributions to osmotic, oxidative, heavy metal, cell wall, and antimicrobial peptide stress resistance, adaptation to antifungal drugs and quorum-sensing molecules, morphogenetic regulation, and virulence (10, 12, 13, 15, 48–51).

The breadth of these roles, together with the observation that Hog1’s outputs differ depending on the nature of the input signal (13), raised an important question about the current paradigm for MAP kinase activation which centers on the phosphorylation of these regulators. What defines the stress specificity of Hog1 outputs? This paradigm of MAP kinase activation appears to require significant tuning. First, our data indicate that strong Hog1 phosphorylation is not essential for accumulation of this MAP kinase in the nucleus (Fig. 1) or for its activation of gene targets in response to specific input signals (Fig. 2). We note that while strong Hog1 phosphorylation is not required for nitrosative-stress gene induction, at least basal phosphorylation of this MAP kinase is essential for nitrosative-stress adaptation, because a nonphosphorylatable *hog1*^AF^ mutant is sensitive to nitrosative stress (Fig. 1A). We were unable to detect any obvious effects of nitrosative stress upon the physical interaction between Hog1 and its export factor Crm1 by coimmunoprecipitation (see Fig. S5 in the supplemental material). Nevertheless, it is conceivable that while Hog1 phosphorylation might be required for entry into the nucleus, additional forms of posttranslational modification might promote the retention of this MAP kinase in the nucleus.

10.1128/mBio.02229-17.5 FIG S5  Impact of nitrosative stress on the physical interaction between Hog1 and its export factor Crm1. Download FIG S5, TIF file, 0.4 MB. Copyright © 2018 Herrero-de-Dios et al. 2018 Herrero-de-Dios et al. This content is distributed under the terms of the Creative Commons Attribution 4.0 International license.

Second, and most significantly, we show that a MAP kinase can be oxidized in response to nitrosative stress. Several findings support this view. First, Hog1 forms disulfides following exposure to nitrosative stress (Fig. 3A). Second, trioxidation of residue C271 following nitrosative stress was observed directly by proteomic analyses of Hog1 tryptic peptides (Fig. S2). The conservative C271S mutation was predicted to not affect the tertiary structure of Hog1 significantly (Fig. S3), it did not affect Hog1 levels (Fig. 4C and S4 and 4C), and it did not compromise the role of Hog1 in stress adaptation, because *HOG1*^C271S^ cells were as resistant as wild-type cells to nitrosative, oxidative, and osmotic stresses (Fig. 4B). However, dramatic changes in Hog1 phosphorylation dynamics were observed in *HOG1*^C271S^ cells (Fig. 4C), but these did not appear to affect the induction of Hog1 target genes (Fig. 4D). We note that, unlike the situation in *S. cerevisiae* and *Cryptococcus neoformans* (52, 53), hyperphosphorylation of Hog1 is not lethal in *C. albicans* (65).

Third, we show that specific, evolutionarily conserved cysteine residues (C156 and C161) influence the nitrosative-stress outputs of Hog1. The induction of key stress genes (*YHB1* and *TRR1*) was compromised in response to nitrosative, but not oxidative, stress in *HOG1*^C156S C161S^ cells but not *HOG1*^C271S^ cells (Fig. 4D). Taken together, the data suggest that alternative Hog1 posttranslational modifications, other than TGY phosphorylation, contribute to the regulation of stress-specific outputs for this MAP kinase.

In the general context of MAP kinase signaling, our data indicate that the redox state of a fungal MAP kinase is altered in response to nitrosative stress and that specific cysteine residues in this MAP kinase promote the stress specificity of its outputs. Posttranslation modifications other than phosphorylation have been shown to modulate the activity and specificity of other MAP kinases. For example, the transcriptional outputs of Sty1 in *S. pombe* are altered by oxidation in response to oxidative stress (40), and JNK1 transcription is downregulated in macrophages by *S*-nitrosylation (38). Extending to other types of regulatory network, ROS-mediated oxidation activates AP-1-like transcription factors in evolutionarily divergent yeasts (19, 23, 54), and the oxidation of NF-κB downregulates its transcriptional activity during anti-*Candida* inflammatory responses (55). In the context of fungal infection, our data indicate that Hog1 cysteine residues moderate outputs that affect the resistance of *C. albicans* to phagocytic killing (Fig. 5A) and its ability to cause systemic infection (Fig. 5B). We conclude that stress-specific posttranslational modifications, other than the canonical TGY phosphorylation, occur on the Hog1 stress-activated protein kinase (SAPK) of a major human pathogen and propose this as a key mechanism to drive the stress-specific outputs of this SAPK.

## MATERIALS AND METHODS

### Strain construction and culture conditions.

Strains used in this study are listed in Table S1 in the supplemental material. *C. albicans* was routinely grown at 30°C and 200 rpm in yeast extract-peptone-dextrose (YPD) (56) or in Tris-buffered YPD medium (YPDT; pH 7.4) as described previously (29). Overnight cultures were diluted into fresh YPD or YPDT to an optical density at 600 nm (OD_600_) of 0.2 and incubated until they reached an OD_600_ of 0.8, whereupon they were subjected to the appropriate treatment. Nitrosative stress was imposed with 5 mM NaNO_2_, 5 mM NaNO_2_ plus 25 mM succinic acid, or DPTA-NONOate at the concentrations specified in the figures. Oxidative stress was applied with 5 mM hydrogen peroxide (H_2_O_2_) and osmotic stress with 1 M NaCl (29).

For stress sensitivity tests, *C. albicans* strains were grown to an OD_600_ of 0.8 and then 10-fold serial dilutions were spotted onto YPD or YPDT plates containing the compounds indicated in the figures. Plates were incubated at 30°C for 48 h.

The *HOG1*^C→S^ mutants were constructed by performing site-directed mutagenesis with the QuikChange site-directed mutagenesis kit by following the manufacturer’s specifications (Agilent, Stockport, United Kingdom). The mutagenesis was performed on the plasmid CIp-C-Hog1HM (10) with the primers listed in Table S1. All of the plasmids generated (CIp-C-Hog1^WT^ [where “WT” indicates “wild type”], CIp-C-Hog1^C156S^, CIp-C-Hog1^C161S^, CIp-C-Hog1^C156S C161S^, CIp-C-Hog1^C271S^) were linearized by digestion with BlpI and integrated into the wild-type *HOG1* allele in JC36 (*hog1Δ/HOG1*) (Table S1) to generate the *HOG1*^WT^, *HOG1*^C156S^, *HOG1*^C161S^, *HOG1*^C156S C161S^, and *HOG1*^C271^ strains (Table S1). The *HOG1* open reading frame in each mutant was fully resequenced to confirm the presence of the appropriate mutation and the absence of any secondary mutations.

### Transcript profiling and analysis.

*C. albicans* wild-type (RM1000+CIp20) (Table S1) and *hog1Δ* (JC50) cells were grown in YPDT to an OD_600_ of 0.8, exposed to 0 or 2.5 mM DPTA-NONOate for 10 min, and then immediately harvested and frozen at −80°C. RNA was extracted from these cells by using TRIzol reagent (Invitrogen, Paisley, UK) and FastPrep-24 (MP Biomedicals, Luton, UK) according to the manufacturer’s instructions. The RNA was treated with DNase (Turbo DNase; Ambion, Banchory, UK) and assessed using an Agilent 2100 Bioanalyzer with RNA 6000 nanokits (Agilent). Samples with an RNA integrity number (RIN) of >8 were used for RNA sequencing.

RNA sequencing was performed using an Ion Torrent Proton sequencer. Raw fastq files were processed through FastQC (v. 10.1), Trim Galore (v. 3.1), Samtools (v. 1.19), STAR aligner (v. 2.4), and HTSeq (v. 5.4). Genome alignments were generated against the C_albicans_SC5314_version_A21-s02-m09-r08 chromosome file from the Candida Genome Database (http://www.candidagenome.org) (57). Gene expression analysis was performed using Partek Genomics Suite software, v. 6.6, using a log_2_ data transformation. Gene ontology (GO) term analysis was performed through the Candida Genome Database GO Term Finder and the Cytoscape v. 3 Clue GO plug-in (58). Network construction was performed with cytoscape v.3 freeware (59). A statistical comparison among GO term enrichment percentages was performed with GraphPad Prism (v. 6) using Student’s *t* test for two-tailed data. Data from three independent biological replicates for each condition are available at EBI (https://www.ebi.ac.uk/arrayexpress/) under accession number E-MTAB-5990.

The levels of specific transcripts were measured by qRT-PCR with a Roche Light Cycler 480 II, in triplicate, using independent biological replicates (60). The primers are described in Table S1. The data were expressed relative to the internal *ACT1* mRNA control and then normalized against the transcript levels in unstressed cells. Results were statistically analyzed using one-way analysis of variance (ANOVA) and multiple-comparison and post-Dunnett’s test.

### Hog1 localization.

*C. albicans* cells expressing YFP-tagged Hog1 (JC63) (Table S1) were grown to exponential phase in YPD or YPDT, exposed to the relevant stress, collected after 10 min, fixed in 3.7% paraformaldehyde, and prepared as described previously (10). Nuclei were localized by staining with DAPI (4′,6-diamidino-2-phenylindole), and YFP fluorescence was captured using a DeltaVision Core microscope (Applied Precision, Issaquah, WA). Data from one of three independent experiments are shown; all displayed similar effects. Quantification was performed using ImageJ 1.48 software. Results were analyzed statistically using one-way ANOVA and Dunnett’s multiple-comparison test on at least 10 cells per condition.

### Hog1 phosphorylation and redox state.

To examine Hog1 phosphorylation, exponentially growing *C. albicans* cells were exposed to the stated stress, protein extracts were prepared at the times indicated in the figures, and these extracts were subjected to Western blotting using previously described protocols (10). Phosphorylated Hog1 was detected using a phospho-p38 antibody (Thr180/Tyr182 number 9211; Cell Signalling Technology, Leiden, The Netherlands), total Hog1 was examined with an anti-Hog1 antibody (y-215, sc-9079; Santa Cruz Biotechnology, Heidelberg, Germany), and actin was analyzed with an antiactin antibody (A5060; Sigma-Aldrich, Dorset, UK). The secondary antibody was horseradish peroxidase-linked anti-rabbit IgG (New England Biolabs, Hitchin, UK), which was detected using the ECL SuperSignal West Femto system (Thermo Fisher Scientific). Hog1 phosphorylation was quantified using ImageJ 1.48 software. The data shown are from one of three independent experiments, all of which showed similar effects.

To assay Hog1 redox state, lysates were prepared from 5 × 10^7^ *C. albicans* cells in trichloroacetic acid (TCA) essentially as described previously (23, 40). Briefly, cells were harvested in buffer (100 mM Tris-HCl, pH 8.0, 1% SDS, 1 mM EDTA) containing 20% TCA and snap-frozen. Protein extracts obtained using FastPrep-24 (MP Biomedicals) were washed with acetone, resuspended in the same buffer containing 10% TCA, and washed with acetone. Pellets were treated with 25 mM NEM (Sigma-Aldrich) for 20 min at 25°C in TCA buffer to block free thiols. Proteins were then reprecipitated in TCA, washed in acetone, and then incubated with 50 mM DTT for 1 h at 37°C to reduce existing disulfides. To alkylate the newly exposed thiols, the extracts were reprecipitated with TCA, washed in acetone, and incubated with 25 mM AMS in TCA buffer at 25°C for 30 min and then at 37°C for 5 min. Protein yields were quantified using a BCA protein assay kit (Thermo Fisher Scientific). The alkylated extracts were then subjected to SDS-PAGE, using a loading dye lacking β-mercaptoethanol, and then to Western blotting with rabbit anti-Hog1 antibodies (y-215, sc-9079; Santa Cruz Biotechnology). The data are representative of three independent experiments, all of which showed similar effects.

### Proteomic analysis of Hog1 tryptic peptides.

*C. albicans* JC310 cells expressing TAP-tagged Hog1 and its control with untagged Hog1 (RM1000+Clp20) (Table S1) were grown to an OD_600_ of 0.8 in YPDT, exposed to 0 or 2.5 mM DPTA-NONOate for 10 min, harvested, and washed in chilled distilled water. Cell extracts were prepared in 25 mM Tris-HCl, pH 7.5, 15 mM EGTA, 15 mM MgCl_2_, 0.1% NP-40, 1 mM DTT, 0.1 mM NaF, and 1 mM phenylmethylsulfonyl fluoride (PMSF), supplemented with protease inhibitor tablets (cOmplete, EDTA-free protease inhibitor cocktail; Roche, Welwyn Garden City, UK), and frozen in liquid nitrogen. TAP-tagged Hog1 was purified using Sepharose-IgG beads (GE Healthcare, Little Chalfont, UK), followed by tobacco etch virus (TEV) protease cleavage and purification with calmodulin affinity resin (Agilent Genomics) as described previously (61, 62). Samples were precipitated using a ReadyPrep two-dimensional cleanup kit (Bio-Rad Laboratories Ltd., Watford, UK) and resuspended in 100 µl 50 mM ammonium bicarbonate. Protein digestion was carried out in solution with 25 µl porcine trypsin (20 µg/ml, sequencing grade; Promega UK, Southampton, UK) by using the NPC protocol published by PRIME-XS (http://www.primexs.eu/protocols/Public-Documents/04---Protocols/PRIME-XS-Protocol-NPC-In-Solution-Digestion.pdf/). Peptides were desalted on ZipTip μ-C_18_ stage tips, according to the manufacturer’s instructions (Millipore, Watford, UK), dried by vacuum centrifugation, and dissolved in 10 µl liquid chromatography-mass spectrometry (LC-MS) loading solvent (2% [vol/vol] acetonitrile, 0.15% [vol/vol] formic acid in ultrahigh-quality [UHQ] water). LC-tandem MS (LC-MS/MS) was performed using a Q Exactive Plus/Ultimate 3000RSLC nanoLC-MS/MS system (Thermo Fisher Scientific, Hemel Hempstead, UK) configured for preconcentration onto a PepMap rapid-separation LC (RSLC) C_18_ nano column (50-µm internal diameter [i.d.] by 15 cm). A sample (5 µl) was injected in µlpickup mode and transferred onto the precolumn (C_18_ PepMap 100; 300-µm i.d. by 5 mm) in LC-MS loading solvent at 10 µl/min for 5 min. Peptides were reversed-flushed from the precolumn to the nano column in a multistep gradient of acetonitrile (2 to 64% [vol/vol] in 40 min) in 0.1% formic acid in UHQ water at 300 nl/min. The entire LC program was 69 min long, and MS/MS data were acquired over 5 to 65 min. The Q Exactive Plus was operated in positive polarity using a “top 10” full MS/data-dependent MS/MS method. The full MS scan parameters were a resolution of 70,000, an AGC target of 3e6, a maximum injection time of 50 ms, and a scan range of 375 to 1,750 *m/z*. The data-dependent-MS/MS parameters were an underfill ratio of 4%; a charge exclusion of “unassigned,” 1, 6 to 8, and >8; a peptide match of “preferred”; exclude isotopes set to “on”; and a dynamic exclusion of 40 s. The raw MS data files were processed using Proteome Discoverer v1.4 (Thermo Fisher Scientific) and a workflow incorporating Mascot Server v1.3 (Matrix Science, Inc., London, UK). The raw MS data files were processed using Proteome Discoverer v1.4 (Thermo Fisher Scientific) and a workflow incorporating Mascot Server v2.5 (Matrix Science, Inc., London, UK). The Mascot database was installed from the file <C_albicans_SC5314_A22_current_orf_trans_all.fasta> downloaded from the Candida Genome Database (http://www.candidagenome.org; date stamp, 19 February 2016). The Mascot database search parameters were as follows: a peptide tolerance of 10 ppm, a fragment ion tolerance of 20 milli-mass units, maximum missed cleavages of 1, and the dynamic modifications carbamidomethyl (C), oxidation (M), oxidation (C), dioxidation (C), trioxidation (C), and nitrosyl (C).

### Structural modeling of Hog1.

Structural models of wild-type and mutant versions of *C. albicans* Hog1 were calculated using the structural predictions tool Phyre2 Web portal (63). Appropriate sequence files, including the relevant mutated residues, were input, and an intensive search was carried out using all other default parameters. In each case, the highest-ranking model, based on confidence and percentage identity as calculated by Phyre2, was then used to assess overall structural features. Models were superimposed and analyzed in PyMOL (PyMOL Molecular Graphics System, version 1.8; Schrödinger, LLC). Several structures of the human homologue p38 are available in the Protein Data Bank (PDB), mostly with bound inhibitors and/or ligands. Only two apo structures are available, and the highest-quality model (PDB accession number 1R39) was chosen for structural comparison (64).

### Intracellular ROS levels.

Exponentially growing *C. albicans* cells were treated with 20 µM dihydroethidium (DHE; Thermo Fisher Scientific) and incubated at 30°C for 1 h in darkness. Cells were washed twice with phosphate-buffered saline (PBS) and subjected to fluorescence-activated cell sorting using a BD LSR II flow cytometer (BD Biosciences, Oxford, UK). The data presented, which were analyzed using FlowJo software version 10.0.8 (FlowJo, LLC), are from one of three independent experiments, all of which showed similar effects. The emission of red fluorescence by DHE (612 nm) was dependent on the presence of cells and not mediated by DPTA-NONOate alone.

### Phagocytic-killing assays.

Polymorphonuclear granulocytes (PMNs) were isolated from the blood of healthy donors, obtained according to protocols approved by the College Ethics Review Board, University of Aberdeen (protocol number CERB/2012/11/676). PMNs were isolated with Hitopaque-1119 and Hitopaque-1077 (Sigma-Aldrich) according to the manufacturer’s instructions. PMNs were suspended in RPMI 1640 (Sigma-Aldrich) containing 10% heat-inactivated fetal bovine serum (hiFBS) and adjusted to 10^6^ cells/ml. Exponential *C. albicans* cells were washed three times in PBS, resuspended in 1 ml RPMI 1640, 10% hiFBS, and adjusted to 10^5^ cells/ml. *C. albicans* cells were mixed with PMNs in a ratio of 1:10 (yeast cells to phagocytes) and incubated for 2 h at 37°C in RPMI 1640, 10% hiFBS, 5% CO_2_. The PMNs were then lysed with 0.25% SDS and treated with DNase I (Invitrogen), and *C. albicans* cell viability was determined via counting CFU. Data from three independent replicate experiments, each of which included 2 technical replicates, were analyzed in PraphPad Prism 6 using one-way ANOVA and Tukey’s multiple-comparison test.

### Virulence assays.

The levels of virulence of *C. albicans* strains were compared using the *Galleria mellonella* model of systemic infection. Yeast cells grown in YPD medium at 30°C were harvested, washed, and resuspended PBS. Cells (10^5^) were inoculated into the last proleg of a wax moth larva (20 larvae per *C. albicans* strain). Control groups of larvae received no injection or PBS alone. Larvae were incubated at 37°C, and survival was monitored for 7 days. The data presented, which were analyzed using PraphPad Prism 6 and compared using the log-rank (Mantel-Cox) test, are from one of two independent experiments, all of which showed similar effects.

### Data availability.

The RNA sequencing data set is available in EBI (https://www.ebi.ac.uk/arrayexpress/) under accession number E-MTAB-5990. Other data that support the findings of this study are available from the corresponding author upon reasonable request.

## References

[1] Brewster JL, de Valoir T, Dwyer ND, Winter E, Gustin MC 1993 An osmosensing signal transduction pathway in yeast. Science 259:1760–1763. doi:10.1126/science.7681220. 7681220

[2] Han J, Lee JD, Bibbs L, Ulevitch RJ 1994 A MAP kinase targeted by endotoxin and hyperosmolarity in mammalian cells. Science 265:808–811. doi:10.1126/science.7914033. 7914033

[3] Galcheva-Gargova Z, Dérijard B, Wu IH, Davis RJ 1994 An osmosensing signal transduction pathway in mammalian cells. Science 265:806–808. doi:10.1126/science.8047888. 8047888

[4] Mizoguchi T, Ichimura K, Shinozaki K 1997 Environmental stress response in plants: the role of mitogen-activated protein kinases. Trends Biotechnol 15:15–19. doi:10.1016/S0167-7799(96)10074-3. 9032988

[5] Ghosh AS, Ray D, Dutta S, Raha S 2010 EhMAPK, the mitogen-activated protein kinase from *Entamoeba histolytica* is associated with cell survival. PLoS One 5:e13291. doi:10.1371/journal.pone.0013291. 20949043 PMC2951911

[6] Takekawa M, Posas F, Saito H 1997 A human homolog of the yeast Ssk2/Ssk22 MAP kinase kinase kinases, *MTK1*, mediates stress-induced activation of the p38 and JNK pathways. EMBO J 16:4973–4982. doi:10.1093/emboj/16.16.4973. 9305639 PMC1170132

[7] Klipp E, Nordlander B, Krüger R, Gennemark P, Hohmann S 2005 Integrative model of the response of yeast to osmotic shock. Nat Biotechnol 23:975–982. doi:10.1038/nbt1114. 16025103

[8] Mettetal JT, Muzzey D, Gómez-Uribe C, van Oudenaarden A 2008 The frequency dependence of osmo-adaptation in *Saccharomyces cerevisiae*. Science 319:482–484. doi:10.1126/science.1151582. 18218902 PMC2916730

[9] de Nadal E, Ammerer G, Posas F 2011 Controlling gene expression in response to stress. Nat Rev Genet 12:833–845. doi:10.1038/nrg3055. 22048664

[10] Smith DA, Nicholls S, Morgan BA, Brown AJP, Quinn J 2004 A conserved stress-activated protein kinase regulates a core stress response in the human pathogen *Candida albicans*. Mol Biol Cell 15:4179–4190. doi:10.1091/mbc.E04-03-0181. 15229284 PMC515350

[11] Enjalbert B, MacCallum DM, Odds FC, Brown AJP 2007 Niche-specific activation of the oxidative stress response by the pathogenic fungus Candida albicans. Infect Immun 75:2143–2151. doi:10.1128/IAI.01680-06. 17339352 PMC1865731

[12] Alonso-Monge R, Navarro-García F, Molero G, Diez-Orejas R, Gustin M, Pla J, Sánchez M, Nombela C 1999 Role of the mitogen-activated protein kinase Hog1p in morphogenesis and virulence of *Candida albicans*. J Bacteriol 181:3058–3068. 10322006 10.1128/jb.181.10.3058-3068.1999 PMC93760

[13] Enjalbert B, Smith DA, Cornell MJ, Alam I, Nicholls S, Brown AJP, Quinn J 2006 Role of the Hog1 stress-activated protein kinase in the global transcriptional response to stress in the fungal pathogen *Candida albicans*. Mol Biol Cell 17:1018–1032. doi:10.1091/mbc.E05-06-0501. 16339080 PMC1356608

[14] da Silva Dantas A, Patterson MJ, Smith DA, MacCallum DM, Erwig LP, Morgan BA, Quinn J 2010 Thioredoxin regulates multiple hydrogen peroxide-induced signaling pathways in *Candida albicans*. Mol Cell Biol 30:4550–4563. doi:10.1128/MCB.00313-10. 20679492 PMC2950526

[15] Cheetham J, MacCallum DM, Doris KS, da Silva Dantas A, Scorfield S, Odds F, Smith DA, Quinn J 2011 MAPKKK-independent regulation of the Hog1 stress-activated protein kinase in *Candida albicans*. J Biol Chem 286:42002–42016. doi:10.1074/jbc.M111.265231. 21994942 PMC3234903

[16] Brown GD 2011 Innate antifungal immunity: the key role of phagocytes. Annu Rev Immunol 29:1–21. doi:10.1146/annurev-immunol-030409-101229. 20936972 PMC3434799

[17] Jamieson DJ, Stephen DW, Terrière EC 1996 Analysis of the adaptive oxidative stress response of *Candida albicans*. FEMS Microbiol Lett 138:83–88. doi:10.1111/j.1574-6968.1996.tb08139.x. 8674975

[18] Nikolaou E, Agrafioti I, Stumpf M, Quinn J, Stansfield I, Brown AJP 2009 Phylogenetic diversity of stress signalling pathways in fungi. BMC Evol Biol 9:44. doi:10.1186/1471-2148-9-44. 19232129 PMC2666651

[19] Alarco AM, Raymond M 1999 The bZip transcription factor Cap1p is involved in multidrug resistance and oxidative stress response in *Candida albicans*. J Bacteriol 181:700–708. 9922230 10.1128/jb.181.3.700-708.1999 PMC93433

[20] Zhang XT, de Micheli M, Coleman ST, Sanglard D, Moye-Rowley WS 2000 Analysis of the oxidative stress regulation of the *Candida albicans* transcription factor, Cap1p. Mol Microbiol 36:618–629. doi:10.1046/j.1365-2958.2000.01877.x. 10844651

[21] Alonso-Monge R, Navarro-García F, Román E, Negredo AI, Eisman B, Nombela C, Pla J 2003 The Hog1 mitogen-activated protein kinase is essential in the oxidative stress response and chlamydospore formation in *Candida albicans*. Eukaryot Cell 2:351–361. doi:10.1128/EC.2.2.351-361.2003. 12684384 PMC154845

[22] Patterson MJ, McKenzie CG, Smith DA, da Silva Dantas A, Sherston S, Veal EA, Morgan BA, Maccallum DM, Erwig LP, Quinn J 2013 Ybp1 and Gpx3 signaling in *Candida albicans* govern hydrogen peroxide-induced oxidation of the Cap1 transcription factor and macrophage escape. Antioxid Redox Signal 19:2244–2260. doi:10.1089/ars.2013.5199. 23706023 PMC3869436

[23] Kos I, Patterson MJ, Znaidi S, Kaloriti D, da Silva Dantas A, Herrero-de-Dios CM, d’Enfert C, Brown AJP, Quinn J 2016 Mechanisms underlying the delayed activation of the Cap1 transcription factor in *Candida albicans* following combinatorial oxidative and cationic stress important for phagocytic potency. mBio 7:e00331. doi:10.1128/mBio.00331-16. 27025253 PMC4817257

[24] Arana DM, Alonso-Monge R, Du C, Calderone R, Pla J 2007 Differential susceptibility of mitogen-activated protein kinase pathway mutants to oxidative-mediated killing by phagocytes in the fungal pathogen *Candida albicans*. Cell Microbiol 9:1647–1659. doi:10.1111/j.1462-5822.2007.00898.x. 17346314

[25] Miramón P, Dunker C, Windecker H, Bohovych IM, Brown AJP, Kurzai O, Hube B 2012 Cellular responses of *Candida albicans* to phagocytosis and the extracellular activities of neutrophils are critical to counteract carbohydrate starvation, oxidative and nitrosative stress. PLoS One 7:e52850. doi:10.1371/journal.pone.0052850. 23285201 PMC3528649

[26] Chiranand W, McLeod I, Zhou H, Lynn JJ, Vega LA, Myers H, Yates JR III, Lorenz MC, Gustin MC 2008 *CTA4* transcription factor mediates induction of nitrosative stress response in *Candida albicans*. Eukaryot Cell 7:268–278. doi:10.1128/EC.00240-07. 18083829 PMC2238162

[27] Ullmann BD, Myers H, Chiranand W, Lazzell AL, Zhao Q, Vega LA, Lopez-Ribot JL, Gardner PR, Gustin MC 2004 Inducible defense mechanism against nitric oxide in *Candida albicans*. Eukaryot Cell 3:715–723. doi:10.1128/EC.3.3.715-723.2004. 15189992 PMC420131

[28] Hromatka BS, Noble SM, Johnson AD 2005 Transcriptional response of *Candida albicans* to nitric oxide and the role of the *YHB1* gene in nitrosative stress and virulence. Mol Biol Cell 16:4814–4826. doi:10.1091/mbc.E05-05-0435. 16030247 PMC1237085

[29] Kaloriti D, Tillmann A, Cook E, Jacobsen M, You T, Lenardon M, Ames L, Barahona M, Chandrasekaran K, Coghill G, Goodman D, Gow NAR, Grebogi C, Ho HL, Ingram P, McDonagh A, de Moura APS, Pang W, Puttnam M, Radmaneshfar E, Romano MC, Silk D, Stark J, Stumpf M, Thiel M, Thorne T, Usher J, Yin Z, Haynes K, Brown AJP 2012 Combinatorial stresses kill pathogenic *Candida* species. Med Mycol 50:699–709. doi:10.3109/13693786.2012.672770. 22463109 PMC3483063

[30] Malone JH, Oliver B 2011 Microarrays, deep sequencing and the true measure of the transcriptome. BMC Biol 9:34. doi:10.1186/1741-7007-9-34. 21627854 PMC3104486

[31] Poderoso JJ, Carreras MC, Lisdero C, Riobó N, Schöpfer F, Boveris A 1996 Nitric oxide inhibits electron transfer and increases superoxide radical production in rat heart mitochondria and submitochondrial particles. Arch Biochem Biophys 328:85–92. doi:10.1006/abbi.1996.0146. 8638942

[32] Carreras MC, Franco MC, Peralta JG, Poderoso JJ 2004 Nitric oxide, complex I, and the modulation of mitochondrial reactive species in biology and disease. Mol Aspects Med 25:125–139. doi:10.1016/j.mam.2004.02.014. 15051322

[33] Hess DT, Matsumoto A, Kim SO, Marshall HE, Stamler JS 2005 Protein S-nitrosylation: purview and parameters. Nat Rev Mol Cell Biol 6:150–166. doi:10.1038/nrm1569. 15688001

[34] Switzer CH, Glynn SA, Cheng RY, Ridnour LA, Green JE, Ambs S, Wink DA 2012 S-nitrosylation of EGFR and Src activates an oncogenic signaling network in human basal-like breast cancer. Mol Cancer Res 10:1203–1215. doi:10.1158/1541-7786.MCR-12-0124. 22878588 PMC3463231

[35] Truong TH, Carroll KS 2013 Redox regulation of protein kinases. Crit Rev Biochem Mol Biol 48:332–356. doi:10.3109/10409238.2013.790873. 23639002 PMC4358782

[36] Leichert LI, Dick TP 2015 Incidence and physiological relevance of protein thiol switches. Biol Chem 396:389–399. doi:10.1515/hsz-2014-0314. 25719318

[37] Park HS, Mo JS, Choi EJ 2006 Nitric oxide inhibits an interaction between JNK1 and c-Jun through nitrosylation. Biochem Biophys Res Commun 351:281–286. doi:10.1016/j.bbrc.2006.10.034. 17054907

[38] Park HS, Huh SH, Kim MS, Lee SH, Choi EJ 2000 Nitric oxide negatively regulates c-Jun N-terminal kinase/stress-activated protein kinase by means of S-nitrosylation. Proc Natl Acad Sci U S A 97:14382–14387. doi:10.1073/pnas.97.26.14382. 11121042 PMC18927

[39] Templeton DJ, Aye MS, Rady J, Xu F, Cross JV 2010 Purification of reversibly oxidized proteins (PROP) reveals a redox switch controlling p38 MAP kinase activity. PLoS One 5:e15012. doi:10.1371/journal.pone.0015012. 21085594 PMC2981573

[40] Day AM, Veal EA 2010 Hydrogen peroxide-sensitive cysteines in the Sty1 MAPK regulate the transcriptional response to oxidative stress. J Biol Chem 285:7505–7516. doi:10.1074/jbc.M109.040840. 20061379 PMC2844198

[41] Eaton P 2006 Protein thiol oxidation in health and disease: techniques for measuring disulfides and related modifications in complex protein mixtures. Free Radic Biol Med 40:1889–1899. doi:10.1016/j.freeradbiomed.2005.12.037. 16716890

[42] Burgoyne JR, Eaton P 2009 Transnitrosylating nitric oxide species directly activate type I protein kinase A, providing a novel adenylate cyclase-independent cross-talk to beta-adrenergic-like signaling. J Biol Chem 284:29260–29268. doi:10.1074/jbc.M109.046722. 19726669 PMC2785556

[43] Delaunay A, Pflieger D, Barrault MB, Vinh J, Toledano MB 2002 A thiol peroxidase is an H_2_O_2_ receptor and redox-transducer in gene activation. Cell 111:471–481. doi:10.1016/S0092-8674(02)01048-6. 12437921

[44] Brown AJP, Haynes K, Quinn J 2009 Nitrosative and oxidative stress responses in fungal pathogenicity. Curr Opin Microbiol 12:384–391. doi:10.1016/j.mib.2009.06.007. 19616469 PMC2728829

[45] Brennan M, Thomas DY, Whiteway M, Kavanagh K 2002 Correlation between virulence of *Candida albicans* mutants in mice and *Galleria mellonella* larvae. FEMS Immunol Med Microbiol 34:153–157. doi:10.1111/j.1574-695X.2002.tb00617.x. 12381467

[46] Li DD, Deng L, Hu GH, Zhao LX, Hu DD, Jiang YY, Wang Y 2013 Using *Galleria mellonella*-*Candida albicans* infection model to evaluate antifungal agents. Biol Pharm Bull 36:1482–1487. doi:10.1248/bpb.b13-00270. 23995660

[47] Day AM, Herrero-de-Dios CM, MacCallum DM, Brown AJP, Quinn J 2017 Stress-induced nuclear accumulation is dispensable for Hog1-dependent gene expression and virulence in a fungal pathogen. Sci Rep 7:14340. doi:10.1038/s41598-017-14756-4. 29085028 PMC5662626

[48] San José C, Monge RA, Pérez-Díaz R, Pla J, Nombela C 1996 The mitogen-activated protein kinase homolog HOG1 gene controls glycerol accumulation in the pathogenic fungus Candida albicans. J Bacteriol 178:5850–5852. doi:10.1128/jb.178.19.5850-5852.1996. 8824643 PMC178437

[49] Munro CA, Selvaggini S, de Bruijn I, Walker L, Lenardon MD, Gerssen B, Milne S, Brown AJP, Gow NA 2007 The PKC, HOG and Ca2+ signalling pathways co-ordinately regulate chitin synthesis in *Candida albicans*. Mol Microbiol 63:1399–1413. doi:10.1111/j.1365-2958.2007.05588.x. 17302816 PMC2649417

[50] Yin Z, Stead D, Walker J, Selway L, Smith DA, Brown AJP, Quinn J 2009 A proteomic analysis of the salt, cadmium and peroxide stress responses in Candida albicans and the role of the Hog1 stress-activated MAPK in regulating the stress-induced proteome. Proteomics 9:4686–4703. doi:10.1002/pmic.200800958. 19824012

[51] Prieto D, Román E, Correia I, Pla J 2014 The HOG pathway is critical for the colonization of the mouse gastrointestinal tract by *Candida albicans*. PLoS One 9:e87128. doi:10.1371/journal.pone.0087128. 24475243 PMC3903619

[52] Vendrell A, Martínez-Pastor M, González-Novo A, Pascual-Ahuir A, Sinclair DA, Proft M, Posas F 2011 Sir2 histone deacetylase prevents programmed cell death caused by sustained activation of the Hog1 stress-activated protein kinase. EMBO Rep 12:1062–1068. doi:10.1038/embor.2011.154. 21836634 PMC3185340

[53] Lee JW, Ko YJ, Kim SY, Bahn YS 2011 Multiple roles of Ypd1 phosphotransfer protein in viability, stress response, and virulence factor regulation in *Cryptococcus neoformans*. Eukaryot Cell 10:998–1002. doi:10.1128/EC.05124-11. 21642509 PMC3147412

[54] Toone WM, Morgan BA, Jones N 2001 Redox control of AP-1-like factors in yeast and beyond. Oncogene 20:2336–2346. doi:10.1038/sj.onc.1204384. 11402331

[55] Warnatsch A, Tsourouktsoglou TD, Branzk N, Wang Q, Reincke S, Herbst S, Gutierrez M, Papayannopoulos V 2017 Reactive oxygen species localization programs inflammation to clear microbes of different size. Immunity 46:421–432. doi:10.1016/j.immuni.2017.02.013. 28314592 PMC5965455

[56] Sherman F 1991 Getting started with yeast. Methods Enzymol 194:3–21. doi:10.1016/S0076-6879(02)50954-X. 2005794

[57] Inglis DO, Arnaud MB, Binkley J, Shah P, Skrzypek MS, Wymore F, Binkley G, Miyasato SR, Simison M, Sherlock G 2012 The *Candida* genome database incorporates multiple *Candida* species: multispecies search and analysis tools with curated gene and protein information for *Candida albicans* and *Candida glabrata*. Nucleic Acids Res 40:D667–D674. doi:10.1093/nar/gkr945. 22064862 PMC3245171

[58] Bindea G, Mlecnik B, Hackl H, Charoentong P, Tosolini M, Kirilovsky A, Fridman WH, Pagès F, Trajanoski Z, Galon J 2009 ClueGO: a cytoscape plug-in to decipher functionally grouped gene ontology and pathway annotation networks. Bioinformatics 25:1091–1093. doi:10.1093/bioinformatics/btp101. 19237447 PMC2666812

[59] Shannon P, Markiel A, Ozier O, Baliga NS, Wang JT, Ramage D, Amin N, Schwikowski B, Ideker T 2003 Cytoscape: a software environment for integrated models of biomolecular interaction networks. Genome Res 13:2498–2504. doi:10.1101/gr.1239303. 14597658 PMC403769

[60] Kastora SL, Herrero-de-Dios C, Avelar GM, Munro CA, Brown AJP 2017 Sfp1 and Rtg3 reciprocally modulate carbon source-conditional stress adaptation in the pathogenic yeast *Candida albicans*. Mol Microbiol 105:620–636. doi:10.1111/mmi.13722. 28574606 PMC5575477

[61] Kaneko A, Umeyama T, Hanaoka N, Monk BC, Uehara Y, Niimi M 2004 Tandem affinity purification of the *Candida albicans* septin protein complex. Yeast 21:1025–1033. doi:10.1002/yea.1147. 15449307

[62] Blackwell C, Brown JD 2009 The application of tandem-affinity purification to *Candida albicans*. Methods Mol Biol 499:133–148. doi:10.1007/978-1-60327-151-6_13. 19152045

[63] Kelley LA, Mezulis S, Yates CM, Wass MN, Sternberg MJ 2015 The Phyre2 web portal for protein modeling, prediction and analysis. Nat Protoc 10:845–858. doi:10.1038/nprot.2015.053. 25950237 PMC5298202

[64] Patel SB, Cameron PM, Frantz-Wattley B, O’Neill E, Becker JW, Scapin G 2004 Lattice stabilization and enhanced diffraction in human p38 alpha crystals by protein engineering. Biochim Biophys Acta 1696:67–73. doi:10.1016/j.bbapap.2003.09.009. 14726206

[65] Day AM, Smith DA, Ikeh MAC, Haider M, Herrero-de-Dios CM, Brown AJP, Morgan BA, Erwig LP, MacCallum DM, Quinn J 2017 Blocking two-component signalling enhances *Candida albicans* virulence and reveals adaptive mechanisms that counteract sustained SAPK activation. PLOS Pathog 13:e1006131. doi:10.1371/journal.ppat.1006131. 28135328 PMC5300278

