# Supplementary figures and images for: Redox Regulation, Rather than Stress-Induced Phosphorylation, of a Hog1 Mitogen-Activated Protein Kinase Modulates Its Nitrosative-Stress-Specific Outputs
Source: mBio. 2018 Mar 27;9(2):e02229-17. doi: 10.1128/mBio.02229-17 (PMC5874921; doi:10.1128/mBio.02229-17)

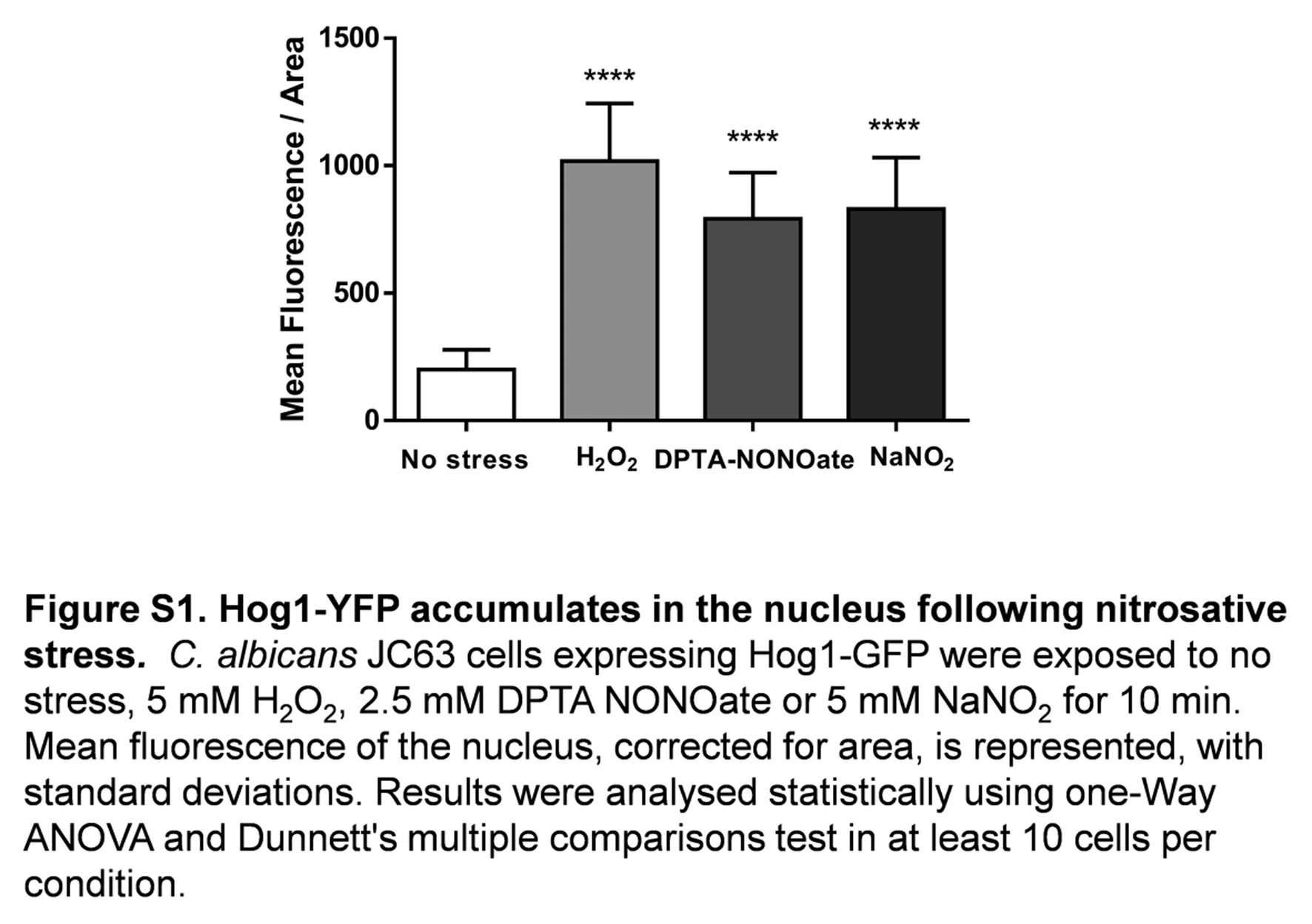

Supplement: FIG S1 [file mbo002183795sf1.tif]

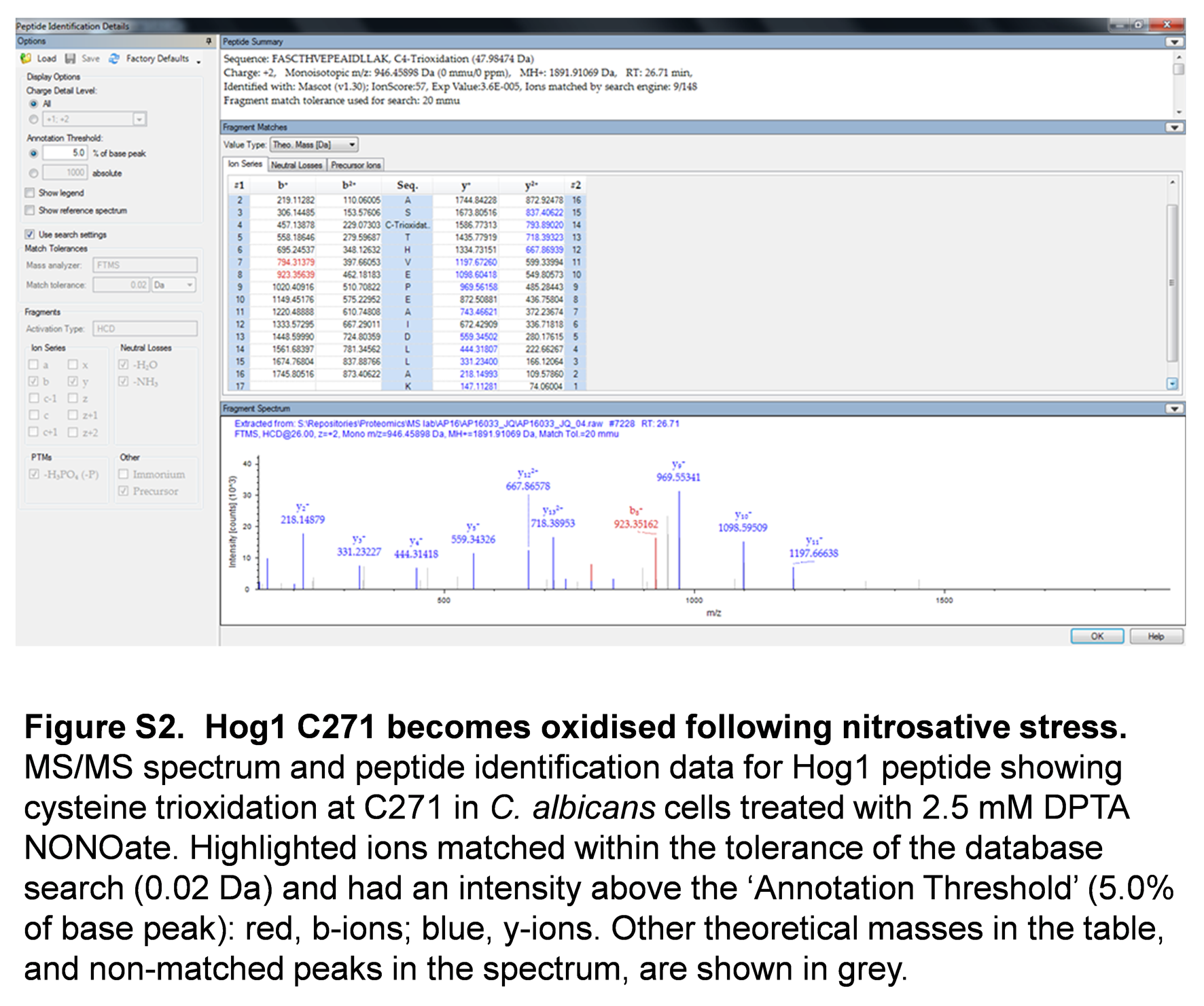

Supplement: FIG S2 [file mbo002183795sf2.tif]

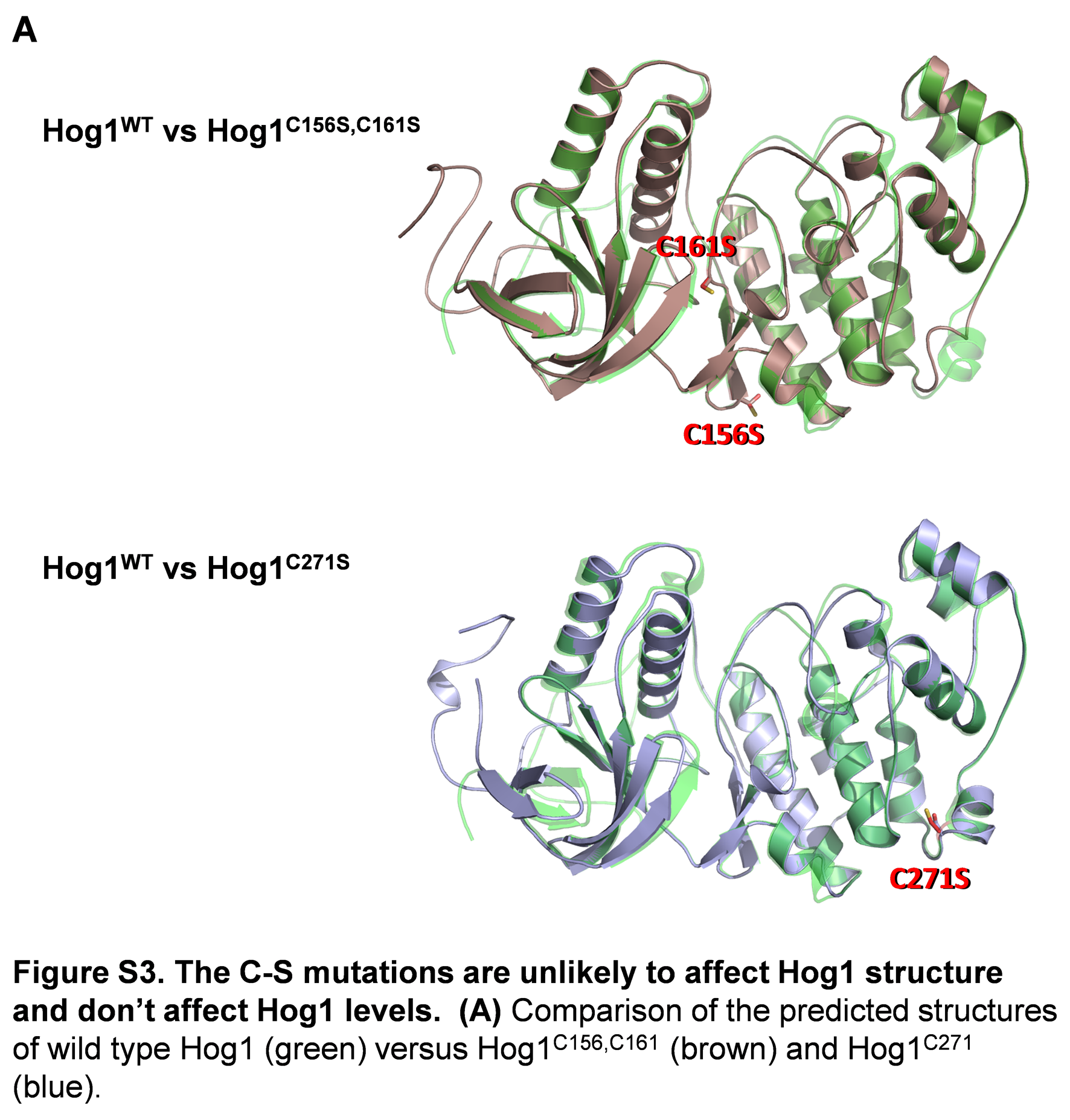

Supplement: FIG S3 [file mbo002183795sf3.tif]

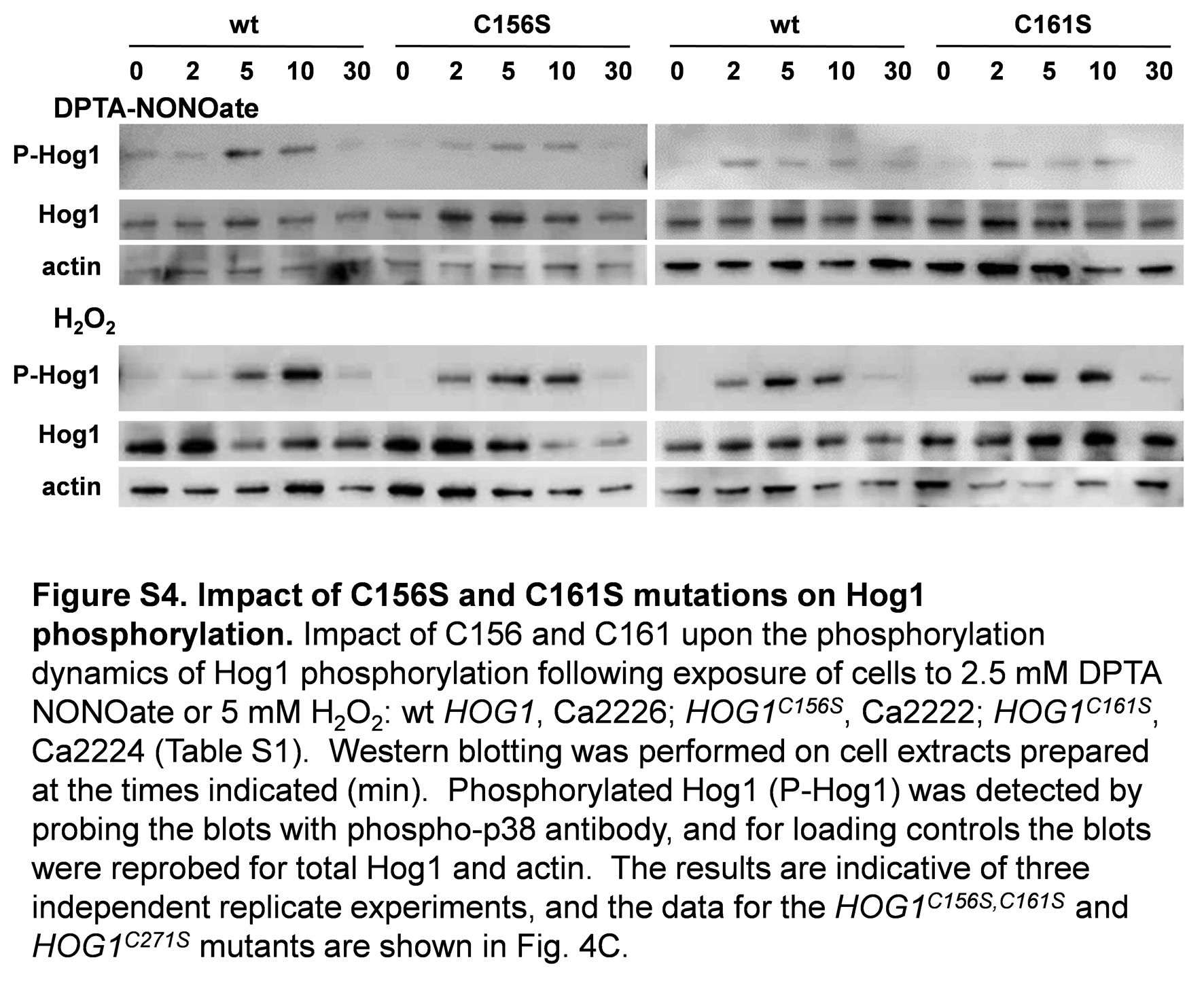

Supplement: FIG S4 [file mbo002183795sf4.tif]

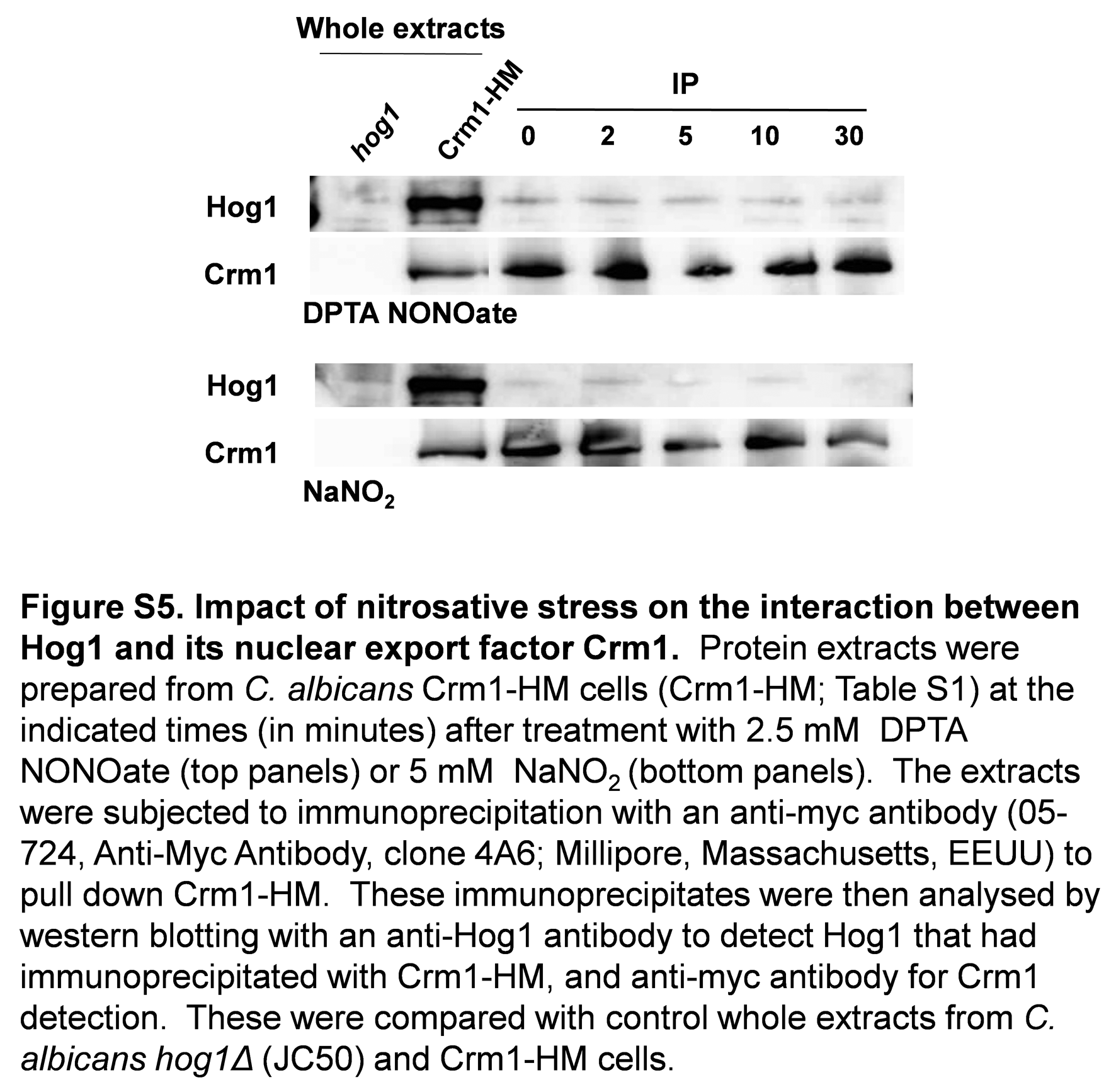

Supplement: FIG S5 [file mbo002183795sf5.tif]
