# Supplementary material for: Redox Regulation, Rather than Stress-Induced Phosphorylation, of a Hog1 Mitogen-Activated Protein Kinase Modulates Its Nitrosative-Stress-Specific Outputs
Source: mBio. 2018 Mar 27;9(2):e02229-17. doi: 10.1128/mBio.02229-17 (PMC5874921; doi:10.1128/mBio.02229-17)
Supplement: TABLE S1 [file mbo002183795st1.pdf]

**Table S1: Strains and Primers**

## *C. albicans* strains

| Strain | | Parent | Genotype | Source |
|---|---|---|---|---|
| RM1000 | | | *ura3*Δ::*imm434*/*ura3*Δ::*imm434, his1*Δ*::hisG/his1*Δ*::hisG* | Negredo *et al*. 1997 |
| RM1000+CIP20 | WT | RM1000 | *ura3*Δ::*imm434*/*ura3*Δ::*imm434, his1*Δ*::hisG/his1*Δ*::hisG,* CIp20 (*URA3,HIS1*) | Smith *et al.* 2004 |
| BWP17 | WT | RM1000 | *ura3*Δ::*imm434*/*ura3*Δ::*imm434, his1*Δ*::hisG/his1*Δ*::hisG, arg4*Δ*::hisG/arg4*Δ*::hisG* | Wilson *et al*. 1999 |
| JC36 | *hog1/HOG1* | BWP17 | BWP17 + *hog1Δ*::*loxP-ARG4-loxP/HOG1* | Enjalbert *et al.* 2006 |
| JC41 | *hog1/HOG1* | RM1000 | RM1000 + *hog1Δ::loxP-HIS1-loxP* | this study |
| JC50 | *hog1Δ* | RM1000 | RM1000 + *hog1Δ*::*loxP-ura3-loxP/ hog1Δ*::*loxP-HIS1-loxP,* CIp20 (*URA3,HIS1*) | Smith *et al.* 2004 |
| JC52 | *HOG1* | RM1000 | RM1000 + *hog1Δ*::*loxP-ura3-loxP/ hog1Δ*::*loxP-HIS1-loxP,* CIp20-*HOG1* (*URA3,HIS1*) | Smith *et al.* 2004 |
| JC63 | *HOG1-YFP* | RM1000 | RM1000 + *HOG1-YFP-URA3/HOG1-YFP-HIS1* | Smith *et al.* 2004 |
| JC76 | *HOG1*$^{AF}$ | JC36 | BWP17 + *hog1Δ*::*loxP-ARG4-loxP/HOG1*$^{AF}$ | Cheetham *et al.* 2011 |
| JC128 | *cap1Δ* | BWP17 | *ura3::λ imm434/ura3::λ imm434, his1::hisG/his1::hisG, arg4::hisG/arg4::hisG, cap1::hisG/cap1::hisG-URA3-hisG* | Enjalbert *et al.* 2006 |
| JC310 | *HOG1-TAP* | JC41 | RM1000 + *hog1Δ::loxP-HIS1-loxP/HOG1-TAP-URA3* | this study |
| Ca1857 | *cta4Δ* | CAF4-2 | *ura3λ : : imm434/ura3λ : : imm434, cta4Δ : : hisG/cta4Δ : : hisG-URA3-hisG* | Coste *et al.* 2008 |
| Ca2226 | *HOG1*$^{WT}$ | JC36 | BWP17 + *hog1Δ*::*loxP-ARG4-loxP/HOG1*$^{WT}$ | this study |
| Ca2222 | *HOG1*$^{C156S}$ | JC36 | BWP17 + *hog1Δ*::*loxP-ARG4-loxP/HOG1*$^{C156S}$ | this study |
| Ca2224 | *HOG1*$^{C161S}$ | JC36 | BWP17 + *hog1Δ*::*loxP-ARG4-loxP/HOG1*$^{C161S}$ | this study |
| Ca2225 | *HOG1*$^{C156S,C161S}$ | JC36 | BWP17 + *hog1Δ*::*loxP-ARG4-loxP/HOG1*$^{C156S,C161S}$ | this study |
| Ca2216 | *HOG1*$^{C271S}$ | JC36 | BWP17 + *hog1Δ*::*loxP-ARG4-loxP/HOG1*$^{C271S}$ | this study |


Coste, A.T., Ramsdale, M., Ischer, F. & Sanglard, D (2008) Divergent functions of three *Candida albicans* zinc-cluster transcription factors (*CTA4*, *ASG1*and *CTF1*) complementing pleiotropic drug resistance in *Saccharomyces cerevisiae. Microbiol.* **154**, 1491-1501.

Cheetham, J., MacCallum, D.M., Doris, K.S., da Silva Dantas, A., Scorfield, S., Odds, F.C., Smith, D.A. & Quinn, J. (2011) MAPKKK-independent regulation of the Hog1 stress activated protein kinase in *Candida albicans. J. Biol. Chem*. **286**, 42002-42016.

Enjalbert, B., Smith, D.A., Cornell, M.J., Alam, I., Nicholls, S., Brown, A.J.P. & Quinn, J. (2006) Role of the Hog1 stress-activated protein kinase in the global transcriptional response to stress in the fungal pathogen *Candida albicans. Molec. Biol. Cell* **17**, 1018-1032.

Negredo, A., Monteoliva, L., Gil, C., Pla, J. & Nombela, C. (1997) Cloning analysis and one-step disrupton of the *ARG5,6* gene of *Candida albicans*. *Microbiology* **143**, 297-302.

Smith, D.A., Nicholls, S., Morgan, B.A., Brown, A.J.P. & Quinn, J. (2004) A Conserved Stress-Activated Protein Kinase Regulates a Core Stress Response in the Human Pathogen *Candida albicans*. *Molec. Biol. Cell,* **15**, 4179-4190.
Wilson, R.B., Davis, D. & Mitchell, A.P. (1999) Rapid hypothesis testing with *Candida albicans* through gene disruption with short homology regions. *J. Bacteriol.,* **181**, 1868–1874.

## Primers

| Primer Name | Sequence 5' to 3' | Target gene/transcript |
|---|---|---|
| *Site-directed mutagenesis* | | |
| SDM1_HOG1_F | CCAACATCTTGATCAATGAAAACTCCGAC TTGAAAATCTGTGAC | *HOG1* C156S mutation |
| SDM1_HOG1_R | GTCACAGATTTTCAAGTCGGAGTTTTCAT TGATCAAGATGTTGG | |
| SDM2_HOG1_F | CATCTTGATCAATGAAAACTGTGACTTGA AAATCTCCGACTTTGGTCTTGC | *HOG1* C161S mutation |
| SDM2_HOG1_R | GCAAGACCAAAGTCGGAGATTTTCAAGTC ACAGTTTTCATTGATCAAGATG | |
| SDM3_HOG1_F | CCATCTTGATCAATGAAAACTCCGACTTG AAAATCTCCGACTTTGGTCTTGC | *HOG1* C156S & C161S mutations |
| SDM3_HOG1_R | GCAAGACCAAAGTCGGAGATTTTCAAGTC GGAGTTTTCATTGATCAAGATGG | |
| SDM4_HOG1_F | GTGAAAGATTTGCTTCCTCCACTCACGTT GAACCGGAG | *HOG1* C271S mutation |
| SDM4_HOG1_R | CTCCGGTTCAACGTGAGTGGAGGAAGCA AATCTTTCAC | |
| *qRT-PCR* | | |
| L-YHB1 | TGGCGATATTTTGAAAGTTGC | *YHB1* transcript |
| R-YHB1 | CCTTGTCTGTAGCTGGGTCAT | |
| L-TRR1 | TTCAGAAACAACCCATTAGCTG | *TRR1* transcript |
| R-TRR1 | AATGGCTTCTTCACAAGCTGA | |
| L-CAT1 | TGGTTTTATTCTCCGACAGAGG | *CAT1* transcript |
| R-CAT1 | TGACCAGAGTAACCATTCATTTCT | |
| L-CAP1 | GATGATACCAAGAAACTTCACGTAGAT | *CAP1* transcript |
| R-CAP1 | TTTAGGTTCGGTATCGATTGGT | |
| L-ACT1 | ACCACCGGTATTGTTTTGGA | *ACT1* transcript |
| R-ACT1 | AGCGTAAATTGGAACAACGTG | |
