# Supplementary material for: Redox Regulation, Rather than Stress-Induced Phosphorylation, of a Hog1 Mitogen-Activated Protein Kinase Modulates Its Nitrosative-Stress-Specific Outputs
Source: mBio. 2018 Mar 27;9(2):e02229-17. doi: 10.1128/mBio.02229-17 (PMC5874921; doi:10.1128/mBio.02229-17)
Supplement: TABLE S2 [file mbo002183795st2.pdf]

Table S2. Nitrosative stress-regulated genes in *C. albicans* revealed by RNA sequencing

Genes showing a statistically significant increase in expression ($\geq$2-fold) in response to 2.5 mM DPTA-NONOate are shown

The full dataset is available at EBI (www.ebi.ac.uk/arrayexpress) under accession number E-MTAB-5990

GENE			WT+NS/WT Fold Change	p-value	*hog1* +NS/*hog1* Fold Change	p-value	Description
C1_00420W_A	orf19.6065	C1_00420W_A	2.85	0.0003879	1.77699	N/S	RNA polymerase II holoenzyme/mediator subunit; regulate
C1_00490C_A	orf19.6059	TTR1	3.37	0.00016921	2.8574	0.0000477	Putative glutaredoxin; described as a glutathione reductas
C1_00860W_A	orf19.6020	C1_00860W_A	2.26	0.00627058	-1.13445	N/S	Ortholog(s) have Atg8 ligase activity and role in C-terminal
C1_01220C_A	orf19.3310	C1_01220C_A	9.22	0.0157387	3.5	0.00292179	Protein of unknown function; Hap43-repressed; rat cathete
C1_01650W_A	orf19.3355	ISN1	2.62	0.00000555	1.10037	N/S	Putative inosine 5'-monophosphate 5'-nucleotidase; fungal
C1_01810C_A	orf19.4543	UGA2	3.20	0.00201052	1.11246	N/S	Predicted succinate semialdehyde dehydrogenase; predict
C1_02010C_A	orf19.4524	ZCF24	3.31	0.00000164	-1.00429	N/S	Predicted Zn(II)2Cys6 transcription factor; caspofungin ind
C1_02040C_A	orf19.3661	C1_02040C_A	3.48	0.00001	1.77918	N/S	Putative deubiquitinating enzyme; induced by Mnl1 under
C1_02590C_A	orf19.2947	SNZ1	4.06	0.00980716	5.22785	0.00000934	Stationary phase protein; vitamin B synthesis; induced byy
C1_02600W_A	orf19.2948	SNO1	7.07	0.00245964	6.72848	9.55E-07	Protein with a predicted role in pyridoxine metabolism; stat
C1_02810W_A	orf19.2969	RAD16	5.77	0.00077057	6.41972	0.00000721	Ortholog of S. cerevisiae Rad16; a protein that recognizes
C1_03120W_A	orf19.3004	C1_03120W_A	2.09	0.00340401	1.54156	N/S	Ortholog(s) have fungal-type vacuole localization
C1_03270W_A	orf19.3021	C1_03270W_A	3.68	0.00000262	1.0933	N/S	Putative protein of unknown function; Hap43-repressed ge
C1_03380W_A	orf19.3038	TPS2	2.74	0.00822136	2.96957	0.0000741	Trehalose-6-phosphate (Tre6P) phosphatase; mutant heat
C1_03450C_A	orf19.3047	C1_03450C_A	2.27	0.00086753	-1.01962	N/S	Protein kinase-related protein, required for normal sensitiv
C1_03510C_A	orf19.3053	C1_03510C_A	2.66	0.0016667	2.72253	0.00000676	Protein of unknown function; present in exponential and st
C1_03750W_A	orf19.1034	C1_03750W_A	5.65	1.01E-07	3.91857	6.27E-08	Protein with a predicted cytochrome b5-like Heme/Steroid
C1_04320W_A	orf19.1067	GPM2	4.94	0.00000406	2.62676	0.0000271	Putative phosphoglycerate mutase; repressed in hyphae;
C1_04700C_A	orf19.775	C1_04700C_A	4.28	7.49E-07	1.31789	N/S	Ortholog of C. dubliniensis CD36 : Cd36_04450, C. parap
C1_04960C_A	orf19.50	C1_04960C_A	3.20	3.09E-08	1.88943	N/S	Ortholog(s) have role in SCF-dependent proteasomal ubiq
C1_05300C_A	orf19.434	PRD1	3.80	0.00000216	1.86759	N/S	Putative proteinase; transcript regulated by Nrg1, Mig1, an
C1_05540C_A	orf19.411	C1_05540C_A	5.78	4.96E-08	2.14979	0.00094705	Protein similar to GTPase regulators; induced in low iron; t
C1_05560W_A	orf19.410.3	RIB4	2.08	0.00223183	1.22083	N/S	Lumazine synthase (6,7-dimethyl-8-ribityllumazine synthas
C1_06520C_A	orf19.6261	BPH1	5.29	6.26E-09	2.08445	4.04E-07	Ortholog of S. cerevisiae Bph1; a putative ortholog of hum
C1_06780W_A	orf19.6232	NPR1	2.94	0.00606193	2.18565	0.00022913	Predicted serine/threonine protein kinase, involved in regu
C1_06810W_A	orf19.6229	CAT1	12.48	0.00136028	9.28845	0.00022259	Catalase; resistance to oxidative stress, neutrophils, perox
C1_06850W_A	orf19.6225	PCL7	2.26	0.0042756	1.97022	N/S	Putative cyclin-like protein; possible Pho85 cyclin; hyphal
C1_06940C_A	orf19.6214	ATC1	13.03	0.00399237	10.5338	0.0000313	Cell wall acid trehalase; catalyzes hydrolysis of the disacc

C1_07100C_A	orf19.6194	C1_07100C_A	2.05	0.014447	1.07229	N/S	Ortholog of C. dubliniensis CD36 : Cd36_06640, C. paraps
C1_07180W_A	orf19.4449	CCS1	2.75	0.00523765	2.47407	0.00117222	Copper chaperone involved in activation and protection of
C1_07230W_A	orf19.4444	PHO15	4.80	0.0000073	3.62514	0.00000152	4-nitrophenyl phosphatase, possible histone H2A phospha
C1_07350C_A	orf19.4436	GPX3	2.29	0.0058858	1.43827	N/S	Putative glutathione peroxidase involved in Cap1p-depend
C1_07880C_A	orf19.5059	GCS1	2.24	0.0000551	3.79053	1.45E-08	Gamma-glutamylcysteine synthetase; glutathione synthesi
C1_07940W_A	snR67	C1_07940W_A	2.66	0.00431158	1.0661	N/S	C/D box small nucleolar RNA (snoRNA)
C1_07980C_A	orf19.5069	C1_07980C_A	4.00	0.00390517	-1.09524	N/S	Ortholog of S. cerevisiae Sae3; meiosis specific protein inv
C1_08060W_A	orf19.5078	OFR1	2.46	0.00012078	1.82663	N/S	Protein of unknown function; Hap43-repressed gene; rat ca
C1_08070W_A	orf19.5079	CDR4	4.21	0.00017327	3.55913	0.0129845	Putative ABC transporter superfamily; fluconazole, Sfu1, H
C1_08240C_A	orf19.5103	C1_08240C_A	2.23	0.0173332	5.91111	0.0000551	Protein with a predicted phosphoglycerate mutase family d
C1_08430W_A	orf19.388	CAF16	6.78	0.00000355	3.32838	0.00114205	ABC family protein, predicted not to be a transporter; Hap4
C1_08590C_A	orf19.406	ERG1	2.93	5.03E-07	2.18842	0.00000277	Squalene epoxidase, epoxidation of squalene to 2,3(S)-oxi
C1_08620W_A	orf19.4720	CTR2	3.92	0.00022442	3.16514	0.0000917	Putative low-affinity copper transporter of the vacuolar mer
C1_08800W_A	orf19.4738	C1_08800W_A	2.05	0.0156849	2.07941	0.00017587	Ortholog(s) have role in protein folding in endoplasmic retic
C1_08830C_A	orf19.4741	C1_08830C_A	4.22	0.00116438	-1.13636	N/S	Protein of unknown function; C. albicans- and C. dublinien
C1_08840W_A	orf19.4742	C1_08840W_A	2.02	0.0023272	1.57377	N/S	Putative SH3-domain-containing protein
C1_08880W_A	orf19.4747	HEM14	2.35	0.00012927	1.4871	N/S	Putative protoporphyrinogen oxidase; involved in heme bio
C1_08940C_A	orf19.4752	MSN4	4.72	0.00096274	2.31646	0.0000103	Zinc finger transcription factor; similar to S. cerevisiae Msn
C1_08960W_A	snR190	C1_08960W_A	3.07	0.0151197	-1.3163	N/S	C/D box small nucleolar RNA (snoRNA)
C1_08980C_A	orf19.4754	ZWF1	2.68	0.00521713	1.21644	N/S	Glucose-6-phosphate dehydrogenase; antigenic in mice; a
C1_09000W_A	orf19.4756	C1_09000W_A	3.19	0.000863	-1.01775	N/S	Ortholog of S. cerevisiae : YTP1, C. dubliniensis CD36 : C
C1_09010W_A	orf19.4757	NAR1	2.13	0.00491571	2.93952	9.86E-08	Putative cytosolic iron-sulfur (FeS) protein assembly mach
C1_09150W_A	orf19.4773	AOX2	16.43	5.92E-10	7.11	0.00978769	Alternative oxidase; cyanide-resistant respiration; induced
C1_09190C_A	orf19.4777	DAK2	5.28	0.00000606	3.32993	0.0000282	Putative dihydroxyacetone kinase; repressed by yeast-hyp
C1_09240C_A	orf19.4783	C1_09240C_A	2.90	0.0070538	-1.09259	N/S	Protein of unknown function; induced during chlamydospor
C1_09250W_A	orf19.4784	CRP1	3.57	0.00010477	2.51934	N/S	Copper transporter; CPx P1-type ATPase; mediates Cu re
C1_09380W_A	orf19.4800	RIM20	2.77	0.0000975	-1.22524	N/S	Protein involved in the pH response pathway; binds to the
C1_09400C_A	orf19.4802	FTH1	3.41	0.00012123	1.44444	N/S	Protein similar to S. cerevisiae Fth1p, a high affinity iron tra
C1_09520C_A	orf19.4816	C1_09520C_A	4.28	0.00003	3.45091	N/S	Protein of unknown function; induced by nitric oxide
C1_09650W_A	orf19.4828	C1_09650W_A	3.14	0.002978	1.76026	N/S	WD repeat domain protein; Hap43-repressed gene; flow m
C1_10040W_A	orf19.4871	ERO1	2.08	0.00028946	-1.1377	N/S	Ortholog of S. cerevisiae Ero1; role in formation of disulfide
C1_10180C_A	orf19.4887	ECM21	2.42	0.00136272	1.91833	N/S	Predicted regulator of endcytosis of plasma membrane pro
C1_10280C_A	orf19.4898	C1_10280C_A	2.48	0.0166787	2.4817	0.00265584	Putative protein of unknown function; induced by prostagla
C1_10290W_A	orf19.4899	GCA1	2.23	0.00824603	4.7549	8.29E-11	Extracellular/plasma membrane-associated glucoamylase;
C1_10510W_A	orf19.994	C1_10510W_A	2.23	0.0227589	1.1087	N/S	Protein of unknown function; induced by Mnl1 under weak

C1_10700C_A	orf19.2351	NIT3	2.41	0.0123307	2.45911	0.00000624	Putative nitrilase; regulated by Gcn2p and Gcn4p; protein
C1_10740C_A	orf19.2344	ASR1	7.69	0.00074991	2.69416	N/S	Heat shock protein; transcript regulated by cAMP, osmotic
C1_10820C_A	orf19.2335	C1_10820C_A	2.24	8.93E-07	1.52536	N/S	Putative aspartyl aminopeptidase; stationary phase enrich
C1_10930C_A	orf19.2324	UBA4	2.56	0.00000306	3.21477	0.0000886	Putative ubiquitin activating protein; Hap43-repressed; ind
C1_11180C_A	snR64	C1_11180C_A	4.29	0.00127854	-3.98658	0.0021775	C/D box small nucleolar RNA (snoRNA)
C1_11340W_A	orf19.669	PRM1	3.47	0.0000429	2.95833	0.0000107	Putative membrane protein with a predicted role in membr
C1_11470C_A	orf19.656	DPP1	3.78	0.0002488	-1.89815	N/S	Putative diacylglycerol pyrophosphate phosphatase of dia
C1_11530C_A	orf19.1167	C1_11530C_A	3.80	0.00636576	3.19355	0.00141786	Ortholog(s) have sulfonate dioxygenase activity and role i
C1_11580W_A	orf19.1162	C1_11580W_A	5.88	0.00000416	3.27092	8.04E-07	Protein of unknown function; transcript upregulated by ben
C1_11700C_A	orf19.1149	MRF1	16.30	0.0000431	6.65842	5.45E-07	Putative mitochondrial respiratory protein; induced by farn
C1_11850W_A	orf19.5282	C1_11850W_A	6.06	0.00000915	6.05042	0.0059233	Protein of unknown function; Hap43-repressed gene; mRN
C1_12060C_A	orf19.5259	C1_12060C_A	2.14	0.00778676	1.9645	N/S	Ortholog of C. dubliniensis CD36 : Cd36_11290, C. paraps
C1_12070C_A	orf19.5258	C1_12070C_A	3.75	0.00022237	1.73415	N/S	Protein of unknown function; induced by nitric oxide
C1_12240C_A	orf19.5239	C1_12240C_A	2.60	0.00063346	-1.01203	N/S	Predicted alanine-tRNA ligase; oxidative stress-induced vi
C1_12360C_A	orf19.5228	RIB3	8.35	7.32E-11	4.29889	5.93E-09	3,4-Dihydroxy-2-butanone 4-phosphate synthase; homodi
C1_12730W_A	orf19.6349	RVS162	4.00	0.0000255	1.67606	N/S	Protein containing a BAR domain, which is found in protei
C1_12850W_A	orf19.4914.1	BLP1	12.26	0.00017176	1.22886	N/S	Protein of unknown function, serum-induced
C1_12970C_A	orf19.4928	SEC2	2.43	0.00061418	1.87061	N/S	Guanyl-nucleotide exchange factor for the small G-protein
C1_13160W_A	orf19.4943	PSA2	2.14	0.0234111	1.51466	N/S	Mannose-1-phosphate guanyltransferase; Hap43, macrop
C1_13270W_A	orf19.4953	C1_13270W_A	3.46	0.00000364	-1.35587	N/S	Putative ATPase; predicted role in ER-associated protein
C1_13470W_A	orf19.4979	KNS1	8.50	0.0128275	-1.01802	N/S	Protein kinase involved in negative regulation of PolIII tran
C1_13480W_A	orf19.4980	HSP70	17.82	0.00026953	2.8777	0.000094	Putative hsp70 chaperone; role in entry into host cells; hea
C1_14030W_A	orf19.7210	C1_14030W_A	2.97	0.0145203	1.63281	N/S	Protein of unknown function; Spider biofilm induced
C1_14040W_A	orf19.7212	C1_14040W_A	2.37	0.0073347	1.92959	N/S	Phosphorylated protein; homozygous transposon insertion
C1_14180W_A	orf19.7225	C1_14180W_A	3.80	0.00145941	1.65169	N/S	Ortholog of C. dubliniensis CD36 : Cd36_13150, C. paraps
C1_14190C_A	orf19.7227	C1_14190C_A	2.33	0.0187457	2.25581	N/S	Protein phosphatase inhibitor; Hap43-repressed; homozyg
C2_00250W_A	orf19.2107.1	STF2	4.53	0.0012137	6.34232	4.71E-08	Protein involved in ATP biosynthesis; repressed in hyphae
C2_00260C_A	orf19.2107	MUQ1	2.72	0.00348105	2.12253	0.00610934	Putative choline phosphate cytidylyltransferase/phosphoet
C2_00510W_A	orf19.2076	C2_00510W_A	6.06	0.0000103	3.51915	0.0000419	Protein of unknown function; S. pombe ortholog SPAC7D4
C2_00530W_A	orf19.2074	C2_00530W_A	20.33	0.00000263	2.6	N/S	Ortholog of Candida albicans WO-1 : CAWG_03833
C2_00540W_A	orf19.2073	C2_00540W_A	5.50	0.00149681	4.09618	N/S	Protein with a multidrug and toxin extrusion protein domai
C2_00760C_A	orf19.2048	C2_00760C_A	53.99	1.04E-08	13.1195	5.42E-13	Proten of unknown function; transcript positively regulated
C2_00770W_A	orf19.2047	C2_00770W_A	4.45	0.0000679	2.42941	7.74E-07	Putative protein of unknown function; Hap43p-repressed g
C2_00780W_A	orf19.2046	POT1-2	2.80	0.0129419	3.55752	0.0000124	Putative peroxisomal 3-ketoacyl CoA thiolase; Hap43-repr
C2_01270W_A	orf19.1996	CHA1	2.12	0.0029028	2.67391	N/S	Similar to catabolic ser/thr dehydratases; repressed by Rin

C2_02010C_A	orf19.1515	CHT4	2.49	0.00143151	-1.51087	N/S	Chitinase; similar to S. cerevisiae sporulation-specific Cts2
C2_02850W_A	orf19.5820	UGA6	11.56	0.0129927	3.6875	0.00000498	Putative GABA-specific permease; decreased transcription
C2_02860W_A	orf19.5818	SUR2	3.52	0.00000141	2.41499	2.39E-10	Putative ceramide hydroxylase; predicted enzyme of sphin
C2_03030W_A	orf19.5798	LIG4	2.53	0.0000914	1.82825	N/S	DNA ligase; mRNA detected in yeast-form and pseudohyp
C2_03170W_A	orf19.5777	C2_03170W_A	2.60	0.00370609	-1.17172	N/S	Protein of unknown function; F-12/CO2 early biofilm induce
C2_03260W_A	orf19.904	C2_03260W_A	3.41	0.0000558	1.39931	N/S	Ortholog(s) have cytosol, nucleus localization
C2_04010C_A	orf19.822	HSP21	5.89	0.00029174	2.27966	N/S	Small heat shock protein; role in stress response and virul
C2_05060C_A	orf19.3537	C2_05060C_A	48.88	4.51E-09	25.6607	0.00000935	Putative sulfiredoxin; regulated by Tsa1, Tsa1B in minimal
C2_05070W_A	orf19.3538	FRE9	3.96	0.00966446	7.125	0.00162624	Ferric reductase; alkaline induced; ciclopirox olamine; Hap
C2_05700W_A	orf19.6882	OSM1	4.61	0.0100756	3.96683	0.0000445	Putative flavoprotein subunit of fumarate reductase; solubl
C2_06430C_A	orf19.21	C2_06430C_A	2.86	0.0000263	2.1118	0.0000263	Ortholog(s) have role in ethanol metabolic process and mit
C2_06550W_A	orf19.31	C2_06550W_A	3.61	0.00598196	1.75	N/S	Ortholog of C. parapsilosis CDC317 : CPAR2_201960, C.
C2_06600W_A	orf19.35	C2_06600W_A	3.28	0.00000277	1.46549	N/S	Predicted kinase; rat catheter, flow model, Spider biofilm ir
C2_06720W_A	orf19.3150	GRE2	5.27	0.00898827	1.41099	N/S	Putative reductase; Nrg1 and Tup1-regulated; benomyl- ar
C2_06890C_A	orf19.2244	C2_06890C_A	3.53	0.00026319	-1.17895	N/S	Similar to oxidoreductases and to S. cerevisiae Yjr096wp;
C2_06940C_A	orf19.2248	ARE2	6.98	9.31E-10	3.14706	7.39E-09	Acyl CoA:sterol acyltransferase; uses cholesterol and oleo
C2_07060W_A	orf19.2261	C2_07060W_A	2.33	0.0000639	3.8513	0.00000333	Ortholog(s) have RNA binding activity, role in mRNA splici
C2_07070W_A	orf19.2262	C2_07070W_A	12.67	0.0000185	4.93819	0.00000204	Protein similar to quinone oxidoreductases; induced by ber
C2_07140W_A	orf19.2269	C2_07140W_A	2.40	0.00280862	-1.15453	N/S	Putative 3-phosphoserine phosphatase; induced by benom
C2_07570W_A	orf19.1868	RNR22	13.81	6.36E-16	10.3766	1.57E-17	Putative ribonucleoside diphosphate reductase;colony mor
C2_07630C_A	orf19.1862	C2_07630C_A	8.68	1.28E-10	2.84367	0.00015025	Possible stress protein; increased transcription associated
C2_08100W_A	orf19.2175	C2_08100W_A	2.76	0.0000311	2.94563	0.0000394	Putative mitochondrial cell death effector; induced by nitric
C2_08120W_A	orf19.2173	MAF1	2.28	0.00046127	1.29633	N/S	Putative negative regulator of RNA polymerase III; decreas
C2_08130W_A	orf19.2172	ARA1	3.98	0.00228687	1.14254	N/S	D-Arabinose dehydrogenase; dehydro-D-arabinono-1,4-lac
C2_08200W_A	orf19.2165	C2_08200W_A	12.95	3.84E-15	8.54671	1.6E-10	Predicted hydrolase; induced by nitric oxide
C2_08260W_A	orf19.2220	C2_08260W_A	10.50	0.00075391	?	N/S	Protein of unknown function; Hap43-repressed gene; by R
C2_08290C_A	orf19.1354	UCF1	2.94	0.00549309	1.93471	N/S	Upregulated by cAMP in filamentous growth; induced in hig
C2_08300C_A	orf19.1353	C2_08300C_A	4.54	0.0106387	1.47423	N/S	Protein of unknown function; repressed by yeast-hypha sw
C2_08390W_A	orf19.1433	C2_08390W_A	20.66	0.00000264	5.94606	2.55E-08	Protein of unknown function; Hap43-repressed; colony mo
C2_08420W_A	orf19.3639	C2_08420W_A	7.03	3.36E-10	2.62679	7.46E-10	Ortholog(s) have alkylbase DNA N-glycosylase activity and
C2_08860W_A	orf19.223	C2_08860W_A	2.75	0.00030695	1.84927	N/S	Putative serine/threonine protein kinase; Hap43-repressed
C2_09480W_A	orf19.1406	C2_09480W_A	2.80	0.00044412	-1.22066	N/S	Ortholog(s) have DNA-directed DNA polymerase activity, r
C2_09590C_A	orf19.1395	C2_09590C_A	3.83	0.000214	3.26667	N/S	Ortholog(s) have inorganic phosphate transmembrane trar
C2_09710C_A	orf19.1381	C2_09710C_A	4.09	0.00030542	3.82866	0.00000229	Ortholog of S. cerevisiae/S. pombe Lsb5; predicted role in
C2_09860C_A	orf19.1365	C2_09860C_A	3.45	0.0229155	1.5875	N/S	Putative monooxygenase; mutation confers hypersensitivit

C2_10240W_A	orf19.1756	GPD1	2.55	0.00040749	5.9031	5.9031	
C2_10450W_A	orf19.5320	NCE4	2.25	0.00045981	1.24413	N/S	Putative RecQ-mediated genome instability protein; Hap43
C2_10690W_A	orf19.5348	TPS3	4.20	0.00218131	5.03544	0.00014111	Predicted trehalose-phosphate synthase regulatory subuni
C3_00320W_A	orf19.5437	RHR2	6.70	0.00022817	5.47801	0.00304339	Glycerol 3-phosphatase; roles in osmotic tolerance, glycer
C3_00480C_A	orf19.5417	DOT5	2.71	0.019776	3.13966	0.00032789	Putative nuclear thiol peroxidase; alkaline downregulated;
C3_00600W_A	orf19.5399	IFF11	10.30	0.00044379	3.6129	0.00157196	Secreted protein required for normal cell wall structure and
C3_01280W_A	orf19.1723	C3_01280W_A	2.51	0.00580367	1.74118	N/S	Ortholog(s) have role in response to purine-containing com
C3_01540W_A	orf19.1691	C3_01540W_A	3.73	0.00381069	1.52952	N/S	Plasma-membrane-localized protein; filament induced; Hog
C3_01820W_A	orf19.1664	C3_01820W_A	2.16	0.00872579	1.46758	N/S	Protein of unknown function; expression downregulated in
C3_01930W_A	orf19.1655	PXP2	5.11	0.00205986	1.5	N/S	Putative acyl-CoA oxidase; enzyme of fatty acid beta-oxida
C3_02140C_A	orf19.1632	C3_02140C_A	3.13	0.00229171	-1.44326	N/S	Ortholog(s) have cytosol, nucleus localization
C3_02300W_A	orf19.1616	FGR23	3.95	0.00842402	3.49091	0.00189709	Protein of unknown function; repressed by a1/alpha2 in wh
C3_02480C_A	orf19.238	CCP1	7.38	6.62E-07	2.94208	1.65E-07	Cytochrome-c peroxidase N terminus; Rim101, alkaline pH
C3_02610C_A	orf19.251	GLX3	3.33	0.00246348	1.27648	N/S	Glutathione-independent glyoxalase; binds human immuno
C3_03230C_A	orf19.321	C3_03230C_A	3.04	0.00066223	2.11644	0.00230518	Ortholog(s) have L-methionine transmembrane transporter
C3_03720W_A	orf19.6947	GTT11	3.96	0.0005341	1.56453	N/S	Glutathione S-transferase, localized to ER; induced in expo
C3_04060C_A	orf19.2803	HEM13	5.56	0.0000351	4.29228	0.0000272	Coproporphyrinogen III oxidase; antigenic; on yeast cell su
C3_04150W_A	snR17A	C3_04150W_A	2.93	0.0165531	1.15385	N/S	U3 small nucleolar RNA (snoRNA)
C3_04480C_A	orf19.5902	RAS2	3.18	0.00512176	4.21053	0.0000454	Protein similar to S. cerevisiae Ras2; has opposite effects
C3_04550C_A	orf19.5911	CMK1	2.49	0.00477403	1.88506	N/S	Putative calcium/calmodulin-dependent protein kinase II; e
C3_04650W_A	orf19.5925	C3_04650W_A	3.37	0.00016667	-1.5493	N/S	Ortholog of S. cerevisiae : AIM6, C. glabrata CBS138 : CA
C3_04970C_A	orf19.5965	C3_04970C_A	2.42	0.0002441	1.08825	N/S	Ortholog(s) have ubiquitin-ubiquitin ligase activity, role in E
C3_05080W_A	orf19.5978	C3_05080W_A	3.72	0.00017607	1.66071	N/S	Has domain(s) with predicted oxidoreductase activity, trans
C3_05090C_A	orf19.5980	C3_05090C_A	3.26	0.00000137	1.52429	N/S	Protein of unknown function; Hap43-repressed gene
C3_05360C_A	orf19.6973	C3_05360C_A	3.85	0.0161737	1.4328	N/S	ATP-dependent LON protease family member; Hap43-repr
C3_05600W_A	orf19.6995	ATO6	2.96	0.00754666	4.88462	0.00000314	Putative fungal-specific transmembrane protein
C3_05930W_A	orf19.7374	CTA4	6.81	0.00010274	13.3548	2.63E-07	Zn(II)2Cys6 transcription factor; induced by nitric oxide; inc
C3_06140W_A	orf19.7396	C3_06140W_A	3.05	0.00069185	3.34568	2.35E-08	Protein of unknown function; Spider biofilm induced; Hap43
C3_06180C_A	orf19.7417	TSA1	8.00	0.0094723	3	N/S	TSA/alkyl hydroperoxide peroxidase C (AhPC) family prote
C3_06270C_A	orf19.7405	C3_06270C_A	2.50	0.00011649	1.31769	N/S	Ortholog of Rad33; involved in nucleotide excision repair in
C3_06330W_A	orf19.7398.1	TSA1B	12.00	0.00447345	?	N/S	Putative peroxidase; orf19.7398.1 is contig-truncated fragn
C3_06490W_A	orf19.7437	C3_06490W_A	9.36	0.00000749	3.95016	0.00000439	Putative protein of unknown function; Hap43p-repressed g
C3_06860C_A	orf19.6816	C3_06860C_A	3.29	0.00000872	2.63542	0.00000187	Putative xylose and arabinose reductase; flow model biofil
C3_06920W_A	orf19.6809	C3_06920W_A	3.10	0.0000281	1.96308	N/S	Putative phosphomutase-like protein; protein present in ex
C3_07270C_A	orf19.6771	UBI4	2.19	0.0119279	-2.15251	0.0160764	Ubiquitin precursor (polyubiquitin) contains 3 tandem repea

C3_07330W_A	orf19.6758	C3_07330W_A	4.15	0.00073672	2.06875	0.00049518	Predcted glucose 1-dehydrogenase (NADP+); rat catheter
C3_07340W_A	orf19.6757	GCY1	2.77	0.0000704	2.15094	0.00000259	Aldo/keto reductase; mutation confers hypersensitivity to to
C3_07440W_A	orf19.6745	TPI1	2.55	0.0201983	4.06576	0.00088307	Triose-phosphate isomerase; antigenic in mouse/human; r
C3_07490W_A	orf19.6739	C3_07490W_A	2.14	0.00821172	2.02964	0.00314776	Ortholog(s) have phosphopentomutase activity, role in dec
C4_00120W_A	orf19.5635	PGA7	6.30	0.0187149	3.91228	N/S	GPI-linked hyphal surface antigen; induced by ciclopirox ol
C4_00150C_A	orf19.5640	PEX5	3.21	0.0010391	1.4758	N/S	Pex5p family protein; required for PTS1-mediated peroxiso
C4_00450C_A	orf19.5674	PGA10	7.35	0.00651557	17.6429	17.6429	
C4_00580W_A	orf19.4185	C4_00580W_A	2.21	0.00569759	1.33939	N/S	Ortholog(s) have phosphoprotein phosphatase activity, thio
C4_00950C_A	orf19.4699	C4_00950C_A	5.91	0.0000835	1.90068	N/S	Putative phospholipase of patatin family; similar to S. cerev
C4_01620C_A	snR77	C4_01620C_A	5.31	0.0207922	1.34247	N/S	C/D box small nucleolar RNA (snoRNA)
C4_01690C_A	orf19.4624	HRT2	4.49	0.00028323	1.54618	N/S	Protein described as having a role in Ty3 transposition; rep
C4_01760W_A	orf19.4617	C4_01760W_A	4.70	0.00143788	3.3568	0.0000879	Predicted peptide alpha-N-acetyltransferase; flow model bi
C4_01800W_A	orf19.4612	C4_01800W_A	3.29	0.00115741	7.77778	7.77778	
C4_01860C_A	orf19.4607	C4_01860C_A	5.73	0.0177454	-1.21084	N/S	Possible Golgi membrane protein; Hap43-repressed; hyph
C4_01970W_A	orf19.4595	C4_01970W_A	3.83	0.00000172	1.57528	N/S	Ortholog of C. dubliniensis CD36 : Cd36_41860, C. paraps
C4_02110W_A	orf19.4580	C4_02110W_A	2.30	0.00141131	2.02239	0.0102728	Protein of unknown function; Hap43-repressed gene
C4_02330C_A	orf19.2770	C4_02330C_A	26.00	0.018473	1.475	N/S	Ortholog of C. dubliniensis CD36 : Cd36_42220, C. paraps
C4_02360W_A	orf19.2768	AMS1	17.31	0.0000193	3.41001	1.97E-09	Putative alpha-mannosidase; transcript regulated by Nrg1;
C4_02410C_A	orf19.2762	AHP1	9.91	0.00054006	6.41318	6.41E-07	Alkyl hydroperoxide reductase; immunogenic; fluconazole-
C4_02990C_A	orf19.2693	GST2	13.89	2.3E-08	6.1255	4.09E-09	Glutathione S transferase; induced by benomyl and in pop
C4_03000C_A	orf19.2691	C4_03000C_A	5.08	0.0150437	3.84	0.00098978	Planktonic growth-induced gene
C4_03100W_A	orf19.2681	RBT7	8.20	0.00190272	4	0.0143945	Protein with similarity to RNase T2 enzymes; has putative
C4_03200C_A	orf19.2670	C4_03200C_A	2.09	0.00477638	1.44133	N/S	Ortholog(s) have 3-hydroxyacyl-[acyl-carrier-protein] dehy
C4_03370C_A	orf19.3364	C4_03370C_A	6.46	8.31E-08	1.65823	N/S	Ortholog of C. parapsilosis CDC317 : CPAR2_403360, De
C4_03430W_A	orf19.3369	MOH1	8.20	0.00018981	1.36	N/S	Ortholog of S. cerevisiae Moh1, essential for staionary pha
C4_03600C_A	orf19.1314	C4_03600C_A	5.83	4.69E-08	1.81166	N/S	Protein of unknown function; planktonic growth-induced ge
C4_03890W_A	orf19.5045	PTP2	3.61	0.0000127	1.60215	N/S	Predicted protein tyrosine phosphatase; involved in regula
C4_03960W_A	orf19.787.1	C4_03960W_A	7.35	0.00172594	2.79225	0.00017316	Protein of unknown function; ORF added to Assembly 21 b
C4_04710W_A	orf19.3811	GYP1	2.84	0.00073018	1.33801	N/S	Putative Cis-golgi GTPase-activating protein; required for h
C4_05140C_A	orf19.744	GDB1	2.83	0.0168467	1.40065	N/S	Putative glycogen debranching enzyme; expression is regu
C4_05300W_A	orf19.1290	XKS1	4.22	0.000003	2.19103	0.00000225	Putative xylulokinase; Hap43-repressed; induced by prosta
C4_05390W_A	orf19.1796	C4_05390W_A	3.79	5.17E-08	1.2389	N/S	Putative glyoxylate reductase; acts on glyoxylate and hydro
C4_05590W_A	orf19.1240	C4_05590W_A	2.11	0.00990315	2.11673	0.00364535	Ortholog of S. cerevisiae : YPR117W, C. glabrata CBS138
C4_06570C_A	orf19.2877	PDC11	2.44	0.00750591	2.97174	0.00012807	Pyruvate decarboxylase; antigenic; on hyphal not yeast ce
C4_06780C_A	orf19.3131	OYE32	28.45	0.00267034	10.3662	4.54E-07	NAD(P)H oxidoreductase family protein; induced by nitric c

C4_06890W_A	orf19.3122	ARR3	8.22	2E-09	1.89569	N/S	Ortholog of S. cerevisiae Arr3; arsenite transporter of the p
C4_06900W_A	orf19.3121	GST1	105.00	0.00425063	9.11111	0.00020552	Putative glutathione S-transferase; upregulated in the pres
C5_00710W_A	orf19.570	IFF8	3.00	0.00881645	1.22222	N/S	Putative GPI-anchored adhesin-like protein; decreased tra
C5_00750C_A	orf19.577	C5_00750C_A	2.34	0.00145048	1.2006	N/S	Predicted protein tyrosine phosphatase; rat catheter biofiln
C5_00880C_A	orf19.1979	GIT3	2.17	0.00247915	-1.15116	N/S	Glycerophosphocholine permease; white cell specific trans
C5_00930C_A	orf19.1974	TFS1	2.82	0.0000387	1.14008	N/S	Putative carboxypeptidase y inhibitor; transcript regulated
C5_01920C_A	orf19.3180	C5_01920C_A	2.24	0.00333816	-1.01802	N/S	Ortholog(s) have phosphatidylinositol-4,5-bisphosphate 5-
C5_02630C_A	orf19.4279	MNN1	7.98	0.00000131	-2.48571	N/S	Putative alpha-1,3-mannosyltransferase; of the mannosyltr
C5_02690W_A	orf19.4287	C5_02690W_A	17.18	5.14E-07	10.9333	1.62E-09	Putative oxidoreductase; Hap43-repressed gene; clade-as
C5_02710W_A	orf19.4290	TRR1	9.48	0.000002	7.34169	0.00023876	Thioredoxin reductase; regulated by Tsa1/Tsa1B, Hap43;
C5_02860C_A	orf19.4309	GRP2	4.43	0.0000115	1.16237	N/S	Methylglyoxal reductase; regulation associated with azole
C5_02930C_A	orf19.4317	GRE3	2.44	0.00299055	1.00396	N/S	Putative D-xylose reductase; antigenic in murine systemic
C5_03240W_A	orf19.2655	BUB3	2.93	0.00014051	1.69543	N/S	Protein similar to S. cerevisiae Bub3; a kinetochore checkp
C5_03490C_A	orf19.6658	C5_03490C_A	5.54	0.000831	1.98891	N/S	Stationary phase enriched protein; predicted ORF from As
C5_04050W_A	orf19.1285	C5_04050W_A	2.29	0.00022987	1.17976	N/S	Plasma membrane-localized protein of unknown function;
C5_04220W_A	orf19.3905	MRV5	11.00	0.0029055	?	N/S	Planktonic growth-induced gene
C5_04360C_A	orf19.3922	C5_04360C_A	3.99	8.32E-09	1.38958	N/S	Possible pyrimidine 5' nucleotidase; protein present in exp
C5_04370C_A	orf19.3923	PGA37	3.86	0.00090449	1	N/S	Putative GPI-anchored protein; Hap43-repressed; Spider b
C5_04800W_A	orf19.3966	CRH12	2.27	0.00637356	4.136	0.00000654	CRH family cell wall protein; transcript regulated by Nrg1 a
C5_04810W_A	orf19.3967	PFK1	3.79	0.00122316	5.94064	0.0000117	Phosphofructokinase alpha subinit; activated by fructose 2
C5_04870W_A	orf19.3973	C5_04870W_A	3.60	0.0150373	1.376	N/S	Ortholog of Hua1, a zinc finger domain protein with sequer
C5_05200C_A	orf19.4013	C5_05200C_A	3.35	0.0000248	2.3118	0.0000535	Putative protein of unknown function; Hap43p-repressed g
C5_05430W_A	orf19.4041	PEX4	2.77	0.00162612	2.45455	N/S	Putative peroxisomal ubiquitin conjugating enzyme; regula
C5_05450C_A	orf19.4044	MUM2	4.59	0.00363801	3.15667	N/S	Protein similar to S. cerevisiae Mum2, a protein essential f
C5_05480W_A	orf19.4048	DES1	3.27	0.00193473	3.80271	N/S	Putative delta-4 sphingolipid desaturase; planktonic growth
C6_00220C_A	LSU-G1431	C6_00220C_A	2.82	0.0183374	-1.50562	N/S	C/D box small nucleolar RNA (snoRNA)
C6_00750C_A	orf19.3651	PGK1	3.51	0.00023409	4.72816	0.0000792	Phosphoglycerate kinase; localizes to cell wall and cytopla
C6_01180C_A	orf19.125	EBP1	10.03	6.63E-12	6.67595	4.09E-09	NADPH oxidoreductase; interacts with phenolic substrates
C6_01300W_A	orf19.137	C6_01300W_A	2.39	3.54E-07	1.18092	N/S	Putative transferase involved in phospholipid biosynthesis;
C6_01410C_A	orf19.3443	OYE2	3.62	0.000057	2.44008	0.0000765	Putative NAPDH dehydrogenase; induced by nitric oxide;
C6_01420C_A	orf19.3442	C6_01420C_A	2.07	0.00013583	1.5437	N/S	Putative oxidoreductase; Hap43-repressed gene
C6_01510W_A	orf19.3433	OYE23	27.39	0.00460821	5.41779	0.00000168	Putative NAPDH dehydrogenase; induced by nitric oxide,
C6_01650C_A	orf19.3422	FMP27	2.40	0.00171367	2.16475	0.00000575	Putative mitochondrial protein; mRNA binds She3
C6_01750C_A	orf19.3411	C6_01750C_A	2.04	0.0199634	1.3494	N/S	Ortholog(s) have role in cellular bud site selection and cyto
C6_01870C_A	orf19.3395	C6_01870C_A	3.95	0.0000478	4.80139	0.0000055	Predicted MFS membrane transporter, member of the drug

C6_01990W_A	orf19.689	PLB1	32.50	0.0000103	6.75	0.00452305	Phospholipase B; host cell penetration and virulence in mo
C6_02010C_A	orf19.691	GPD2	2.88	0.00207276	3.02136	0.0000252	Surface protein similar to glycerol 3-P dehydrogenase; bin
C6_02030C_A	orf19.3508	C6_02030C_A	4.24	0.00011118	1.48072	N/S	Putative protein of unknown function; stationary phase enr
C6_02420W_A	orf19.3483	C6_02420W_A	8.57	9.5E-10	1.04211	N/S	Putative phosphatidyl glycerol phospholipase C; Plc1-regu
C6_02480W_A	orf19.5517	C6_02480W_A	3.08	0.0000411	1.63575	N/S	Similar to alcohol dehydrogenases; induced by benomyl tr
C6_02500C_A	orf19.5519	GCV1	2.02	0.00026724	-1.25753	N/S	Putative T subunit of glycine decarboxylase; transcript neg
C6_02560W_A	orf19.5525	C6_02560W_A	3.49	0.00026467	2.04427	0.0139683	Putative oxidoreductase; protein levels affected by URA3 e
C6_02580W_A	orf19.5527	C6_02580W_A	2.17	0.00670089	2.5742	0.00012901	Protein with a predicted role in 5.8S rRNA processing; flow
C6_02950C_A	orf19.5573	C6_02950C_A	7.33	0.00128924	-1.27273	N/S	Protein of unknown function; expression downregulated in
C6_03270C_A	orf19.5615	AYR2	3.55	0.00056964	1.86139	N/S	Putative NADPH-dependent 1-acyl dihydroxyacetone phos
C6_03280W_A	orf19.5616	C6_03280W_A	12.00	0.00630919	12	0.00630919	Ortholog of C. dubliniensis CD36 : Cd36_63740, C. paraps
C6_03480W_A	orf19.5713	YMX6	4.14	7.27E-07	2.93388	0.00033088	Putative NADH dehydrogenase; macrophage-downregulat
C6_03610W_A	orf19.5729	FGR17	3.20	1.49E-09	2.53205	7.92E-09	Putative DNA-binding transcription factor; has zinc cluster
C6_03710W_A	orf19.5742	ALS9	4.09	0.0000309	3.76957	0.0057032	ALS family cell-surface glycoprotein; expressed during infe
C7_00350C_A	orf19.7085	C7_00350C_A	2.72	0.0149194	1.42939	N/S	Protein of unknown function; induced in core stress respor
C7_00760C_A	orf19.7043	C7_00760C_A	6.33	0.0001857	2.16667	0.00331853	Ortholog(s) have endoplasmic reticulum localization
C7_00770W_A	orf19.7042	C7_00770W_A	22.45	0.00000694	4.3	0.00000919	Protein of unknown function; induced by benomyl or in an
C7_01150W_A	orf19.6898	C7_01150W_A	3.66	0.00022239	3.06667	0.00000185	Protein similar to S. pombe SPBC1709.16c a predicted arc
C7_01230C_A	orf19.6905	C7_01230C_A	2.35	0.0141509	1.77517	N/S	Ortholog of S. cerevisiae : YNL011C, C. glabrata CBS138
C7_01540W_A	orf19.6574	ALK6	3.60	0.0205254	8.2	0.00000092	Putative cytochrome P-450 of N-alkane-induced detoxifica
C7_01650W_A	orf19.6559	C7_01650W_A	5.18	0.000093	5.83217	8.37E-07	RNA polymerase III transcription initiation factor complex (
C7_01800C_A	orf19.6540	PFK2	2.28	0.0128492	3.99532	0.0000251	Phosphofructokinase beta subunit; fructose 2,6-bisphosph
C7_02220C_A	orf19.6492	C7_02220C_A	2.40	0.00404532	1.04225	N/S	Predicted protein serine/threonine kinase and/or protein ty
C7_02330W_A	orf19.6478	YCF1	2.43	0.00174655	1.61413	N/S	Putative glutathione S-conjugate transporter; MRP/CFTR-s
C7_02450W_A	orf19.6464	C7_02450W_A	2.30	0.00011539	1.38015	N/S	Protein of unknown function; induced upon adherence to p
C7_02520W_A	orf19.6458	C7_02520W_A	3.12	0.0142909	1.17544	N/S	Ortholog of C. dubliniensis CD36 : Cd36_72260, C. paraps
C7_03200C_A	orf19.5136	C7_03200C_A	3.59	0.00019244	2.59099	0.00033276	Putative pyridoxamine 5'-phosphate oxidase; planktonic gr
C7_03240W_A	orf19.5131	C7_03240W_A	2.25	0.00097892	1.6221	N/S	Ortholog of S. cerevisiae Gid7, a GID complex protein; inv
C7_03450C_A	orf19.1331	HSM3	3.49	0.00000104	-1.44444	N/S	Ortholog(s) have role in mismatch repair, proteasome regu
C7_03520W_A	orf19.6684	PNC1	2.07	0.00041219	1.11765	N/S	Putative nicotinamidase, involved in NAD salvage pathway
C7_03580C_A	orf19.6690	C7_03580C_A	10.53	3.6E-08	2.9183	8.24E-11	Protein of unknown function; Hap43-repressed gene
C7_03680W_A	orf19.6703	C7_03680W_A	2.32	0.00200975	1.44118	N/S	Ortholog of C. dubliniensis CD36 : Cd36_73260 and Cand
C7_03780C_A	orf19.7204	C7_03780C_A	13.38	0.00016434	-1.3121	N/S	Ortholog(s) have glyoxysome localization
C7_03860W_A	orf19.7196	C7_03860W_A	6.45	0.00067541	1.20377	N/S	Putative vacuolar protease; upregulated in the presence of
CM_00190C	CaalfMt28	tC(GCA)3mt	7.00	0.00397449	1	N/S	Mitochondrial cysteine tRNA, predicted by tRNAscan-SE;

CR_00090C_A	orf19.7531	CR_00090C_A	5.20	0.00143909	3.27553	0.0000023	Protein of unknown function; stationary phase enriched pro
CR_00560W_A	orf19.7479	NTH1	4.01	0.00061103	3.39763	3.22E-07	Neutral trehalase; hyphal induction in mutant delayed but r
CR_01200W_A	orf19.3234	OYE22	3.10	0.00012058	1.55405	N/S	Putative NADPH dehydrogenase; rat catheter biofilm induc
CR_01400W_A	orf19.2525	LYS12	3.24	0.00558019	2.98639	2.59E-07	Homoisocitrate dehydrogenase; catalyzes 4th step in the a
CR_02000C_A	orf19.2601	HEM1	4.01	0.00206071	5.9438	0.00029474	Putative 5-aminolevulinate synthase; caspofungin represse
CR_02130W_A	orf19.2613	ECM4	2.96	0.00390188	2.09476	0.0000568	Cytoplasmic glutathione S-transferase; regulated by Nrg1,
CR_02170W_A	orf19.2618	MET2	2.01	0.0145787	2.94007	0.0000517	Homoserine acetyltransferase; Hap43p-, Gcn4p-regulated
CR_02180W_A	orf19.2619	PHO113	3.63	0.00158022	1.79646	N/S	Putative constitutive acid phosphatase; Rim101-repressed
CR_02240C_A	orf19.3746	OPT2	3.25	0.00489998	1.95238	N/S	Oligopeptide transporter; induced upon phagocytosis by m
CR_02570C_A	orf19.164	CR_02570C_A	3.11	5.38E-07	2.5614	0.00000596	Ortholog(s) have lipase activity and peroxisomal matrix loc
CR_02650C_A	orf19.2825	DRE2	2.03	0.00165758	1.78152	N/S	Putative cytosolic Fe-S protein assembly protein; a-specifi
CR_02810W_A	orf19.2839	CIRT4B	4.20	0.0000185	3.22171	1.12E-08	Cirt family transposase; transcript repressed in an azole-re
CR_02820W_A	orf19.2841	PGM2	3.63	0.004923	2.11093	0.0000192	Ortholog of S. cerevisiae Pgm2; induced in planktonic cultu
CR_02960W_A	orf19.2853	CR_02960W_A	2.44	0.0182511	1.12871	N/S	Protein of unknown function; rat catheter biofilm induced
CR_03010C_A	orf19.2863.1	ERV1	2.48	0.00842344	2.39768	0.00414976	Predicted component of the mitochondrial intermembrane
CR_03120W_A	orf19.2414	CR_03120W_A	2.80	0.0000493	2.672	0.00036202	Ortholog of S. cerevisiae Mpm1; a mitochondrial intermem
CR_03220C_A	orf19.2401	CR_03220C_A	2.19	0.0141923	-1.0184	N/S	Ortholog(s) have cysteine-type endopeptidase activity
CR_03690W_A	orf19.4368	CR_03690W_A	2.34	0.00031803	2.32051	2.25E-07	Has domain(s) with predicted hydrolase activity and role in
CR_03710C_A	orf19.4370	CR_03710C_A	4.69	0.0000447	5.63636	0.00576434	Protein of unknown function; induced by nitric oxide; oxida
CR_04060C_A	orf19.489	DAP1	6.12	0.00000408	2.46552	0.00646301	Similar to mammalian membrane-associated progesterone
CR_04220C_A	orf19.510	CR_04220C_A	8.40	0.00050603	10.0909	0.00452084	Protein of unknown function; Spider biofilm induced
CR_04350C_A	orf19.524	CR_04350C_A	2.35	0.00154869	1.249	N/S	Ortholog(s) have role in mitotic sister chromatid segregatic
CR_04480C_A	orf19.539	LAP3	8.17	0.00149958	4.12871	2.02E-07	Putative aminopeptidase; positively regulated by Sfu1; clac
CR_04510W_A	orf19.542	HXK2	3.01	0.00124481	3.03111	0.0000126	Hexokinase II; antigenic in humans; repressed by human r
CR_04590C_A	orf19.550	PDX3	5.58	0.00000147	3.7519	7.42E-08	Pyridoxamine-phosphate oxidase; transcript regulated by y
CR_04960C_A	orf19.633	CRG1	11.85	4.68E-13	6.78182	0.0000261	Methyltransferase involved in sphingolipid homeostasis, m
CR_05340C_A	orf19.5288	IFE2	11.99	1.92E-09	6.17025	2.18E-09	Putative alcohol dehydrogenase; yeast-enriched transcript
CR_05440W_A	orf19.3515	CR_05440W_A	2.54	0.00283087	1.84058	N/S	Putative 3-hydroxyanthranilic acid dioxygenase, involved ir
CR_05720W_A	orf19.6640	TPS1	4.43	0.00040234	2.31182	6.23E-07	Trehalose-6-phosphate synthase; role in hyphal growth an
CR_05740C_A	orf19.6638	PTC4	2.06	0.00113206	-1.1303	N/S	Type PP2C serine/threonine phosphatase; localized to mit
CR_06570C_A	orf19.915	CR_06570C_A	5.08	0.0000137	4.27089	6.11E-08	Protein of unknown function; Spider biofilm induced
CR_06730W_A	orf19.707	APG7	3.27	0.00246527	-1.48763	N/S	Ortholog(s) have APG12 activating enzyme activity, APG8
CR_07150W_A	orf19.734	GLK1	11.66	0.0000454	2.80376	0.00000253	Putative glucokinase; transcript regulated upon yeast-hyph
CR_07160C_A	orf19.733	CR_07160C_A	5.80	0.00082084	2.2	0.00345828	Ortholog(s) have endoplasmic reticulum localization
CR_07480W_A	orf19.6117	CR_07480W_A	8.57	0.0000761	2.28807	0.00367946	S. pombe ortholog SPAC5D6.04 is a predicted auxin famil

CR_07490C_A	orf19.6116	GLK4	10.86	0.00000181	2.88812	4.55E-08	Putative glucokinase; decreased expression in hyphae cor
CR_07790C_A	orf19.3707	YHB1	18.50	6.93E-07	7.88384	5.54E-10	Nitric oxide dioxygenase; acts in nitric oxide scavenging/de
CR_08250C_A	orf19.6387	HSP104	2.68	0.0166057	2.18341	N/S	Heat-shock protein; roles in biofilm and virulence; complen
CR_08310C_A	orf19.6398	CR_08310C_A	5.71	0.00550239	4.66667	N/S	S. pombe ortholog SPBC460.04c is a predicted sulfonate/a
CR_08650C_A	orf19.6440	CR_08650C_A	3.60	0.00058678	2.30934	0.00120601	Ortholog(s) have ubiquitin-protein ligase activity, role in ub
CR_08700C_A	orf19.6447	ARF1	2.80	0.000059	1.14286	N/S	ADP-ribosylation factor; probable GTPase involved in intra
CR_09270C_A	orf19.7323	CBP1	2.70	0.0000356	1.36948	N/S	Corticosteroid binding protein; transcription induced at late
CR_09680C_A	orf19.6595	RTA4	2.23	0.016013	-1.125	N/S	Protein similar to S. cerevisiae Rsb1p, involved in fatty acid
CR_09780C_A	orf19.7550	IFA14	5.36	4.31E-07	2.86957	0.0000463	Putative LPF family protein; Plc1-regulated; induced by alp
CR_09940W_A	orf19.7568	CR_09940W_A	3.33	0.00824322	-5	0.0031821	Ortholog of S. cerevisiae : SPS4, C. glabrata CBS138 : CA
CR_10200W_A	orf19.7596	CR_10200W_A	19.55	1.81E-07	7.28571	N/S	Protein with a phosphoglycerate mutase family domain; Ha
CR_10350C_A	orf19.7611	TRX1	5.37	2.62E-07	2.27368	0.00036434	Thioredoxin; involved in response to reactive oxygen speci
CR_10360C_A	orf19.7612	CTM1	3.57	0.00018236	2.02956	0.00068139	Putative cytochrome c lysine methyltransferase; regulated
