# Supplementary material for: Redox Regulation, Rather than Stress-Induced Phosphorylation, of a Hog1 Mitogen-Activated Protein Kinase Modulates Its Nitrosative-Stress-Specific Outputs
Source: mBio. 2018 Mar 27;9(2):e02229-17. doi: 10.1128/mBio.02229-17 (PMC5874921; doi:10.1128/mBio.02229-17)
Supplement: TABLE S3 [file mbo002183795st3.pdf]

Table S3. Comparison of nitrosative stress-induced genes observed in this study with those identified by Hromatka and co-workers (2005)

Nitrosative stress-induced genes from Table 2 in Hromatka *et al.* (2005), who used 1.0 mM DPTA-NONOate, are compared with nitrosative stress-induced genes in this study (>2 fold up-regulated at 10 min with 2.5 mM DPTA-NONOate).
Genes upregulated in both studies (**blue**). Genes upregulated in the Hromatka study, but not our study (pink).

NC = no significant change in expression level (i.e. fold change = -2 < X < 2)

Hromatka *et al.* (2005) *Molec. Biol. Cell* 16, 4814-4826

Gene			Nitrosative Stress-Induced Genes Hromatka *et al.*	This study
orf19.125	C6_01180C_A	EBP1	25.5	10.0
orf19.3132	C4_06770W_A	C4_06770W_A	18.1	-4.3
orf19.2693	C4_02990C_A	GST2	16.7	13.9
orf19.3707	CR_07790C_A	YHB1	15.8	18.5
orf19.1149	C1_11700C_A	MRF1	12.5	16.3
orf19.4773	C1_09150W_A	AOX2	11.6	16.4
orf19.4290	C5_02710W_A	TRR1	11.4	9.5
orf19.2262	C2_07070W_A	C2_07070W_A	10.7	12.7
orf19.3433	C6_01510W_A	OYE23	10.4	27.4
orf19.6398	CR_08310C_A	CR_08310C_A	10.2	5.7
orf19.3443	C6_01410C_A	OYE2	9.9	3.6
orf19.3131	C4_06780C_A	OYE32	8.0	28.5
orf19.7313	CR_09170C_A	SSU1	7.3	NC
orf19.2601	CR_02000C_A	HEM1	6.8	4.0
orf19.5059	C1_07880C_A	GCS1	6.8	2.2
orf19.3120	C4_06910W_A	C4_06910W_A	6.7	10.0
orf19.7374	C3_05930W_A	CTA4	6.2	6.8
orf19.7042	C7_00770W_A	C7_00770W_A	6.0	22.5
orf19.6229	C1_06810W_A	CAT1	6.0	12.5
orf19.3395	C6_01870C_A	C6_01870C_A	5.7	3.9
orf19.3122	C4_06890W_A	ARR3	5.6	8.2
orf19.5785	C2_03110W_A	C2_03110W_A	5.2	1.3
orf19.4816	C1_09520C_A	C1_09520C_A	5.1	4.3
orf19.2356	CR_07060C_A	CRZ2	5.0	NC
orf19.5770	C6_03930W_A	OPT8	4.9	2.5
orf19.5635	C4_00120W_A	PGA7	4.6	6.3
orf19.4370	CR_03710C_A	CR_03710C_A	4.5	4.7
orf19.5517	C6_02480W_A	C6_02480W_A	4.2	3.1
orf19.7091	C7_00310C_A	C7_00310C_A	4.0	NC
orf19.4147	C5_01520C_A	GLR1	3.9	NC
orf19.4720	C1_08620W_A	CTR2	3.8	3.9
orf19.6586	C7_01430C_A	C7_01430C_A	3.6	NC
orf19.7417	C3_06180C_A	TSA1	3.6	8.0
orf19.5674	C4_00450C_A	PGA10	3.2	7.3

orf19.113	C6_01070C_A	CIP1	3.1	NC
orf19.5636	C4_00130W_A	RBT5	3.1	NC
orf19.711	CR_06550C_A	CR_06550C_A	3.1	7.0
orf19.6478	C7_02330W_A	YCF1	3.1	2.4
orf19.1763	C2_10180W_A	IFR1	3.0	5.4
orf19.4757	C1_09010W_A	NAR1	3.0	2.1
orf19.4907	C1_10360C_A	C1_10360C_A	2.8	NC
orf19.8434	C2_04060C_A	CSY1	2.7	NC
orf19.5713	C6_03480W_A	YMX6	2.7	4.1
orf19.5258	C1_12070C_A	C1_12070C_A	2.7	3.8
orf19.7214	C1_14060W_A	C1_14060W_A	2.7	2.1
orf19.4774	C1_09160W_A	AOX1	2.6	NC
orf19.4802	C1_09400C_A	FTH1	2.6	3.4
orf19.3432	C6_01520W_A	C6_01520W_A	2.5	NC
orf19.1343	C7_03320C_A	MED16	2.5	NC
orf19.3803	C4_04770C_A	MNN22	2.5	NC
orf19.6928	C3_03870C_A	SAP9	2.5	NC
orf19.7316	CR_09190C_A	CR_09190C_A	2.5	NC
orf19.4754	C1_08980C_A	ZWF1	2.5	2.7
orf19.239	C3_02490C_A	C3_02490C_A	2.5	-2.2
orf19.5634	C4_00110C_A	FRP1	2.4	NC
orf19.2165	C2_08200W_A	C2_08200W_A	2.4	13.0
orf19.3117	C4_06920C_A	CSA2	2.3	NC
orf19.1027	C1_03820W_A	PDR16	2.3	NC
orf19.6947	C3_03720W_A	GTT11	2.3	4.0
orf19.5604	C6_03170C_A	MDR1	2.3	-3.5
orf19.2175	C2_08100W_A	C2_08100W_A	2.2	2.8
orf19.2179	C2_08050C_A	SIT1	2.2	-2.2
orf19.4150	C5_01560C_A	C5_01560C_A	2.2	-2.3
orf19.7495	CR_00380W_A	CR_00380W_A	2.1	NC
orf19.2995	C1_03040W_A	C1_03040W_A	2.1	-4.2
orf19.3538	C2_05070W_A	FRE9	1.9	4.0
