# Supplementary material for: Redox Regulation, Rather than Stress-Induced Phosphorylation, of a Hog1 Mitogen-Activated Protein Kinase Modulates Its Nitrosative-Stress-Specific Outputs
Source: mBio. 2018 Mar 27;9(2):e02229-17. doi: 10.1128/mBio.02229-17 (PMC5874921; doi:10.1128/mBio.02229-17)
Supplement: TABLE S4 [file mbo002183795st4.pdf]

Table S4. Comparison of Hog1-dependent genes observed in this study with those identified by Enjalbert and co-workers (2006)

Hog1-dependence was defined as a >2-fold decrease in stress gene induction following Hog1 inactivation
(i.e. fold-induction in *hog1* cells/fold-induction in wild type cells)
Yellow = Hog1-dependent gene induction in response to nitrosative, osmotic, or oxidative stress
NI = not induced (≥2-fold) in response to the stress
Enjalbert *et al.* (2006) Molec. Biol. Cell 17, 1018-1032

GENE		HOG1 DEPENDENCE NITROSATIVE Stress (This study)	OSMOTIC Stress (Enjalbert *et al* 2006)	OXIDATIVE Stress (Enjalbert *et al* 2006)
C1_00420W_A	C1_00420W_A	0.35	NI	NI
C1_00860W_A	C1_00860W_A	0.44	NI	NI
C1_01220C_A	C1_01220C_A	0.38	NI	NI
C1_01360C_A	C1_01360C_A	NI	NI	0.31
C1_01650W_A	ISN1	0.38	NI	0.72
C1_01810C_A	UGA2	0.31	NI	NI
C1_02010C_A	ZCF24	0.30	NI	NI
C1_02040C_A	C1_02040C_A	0.29	0.40	1.16
C1_03120W_A	C1_03120W_A	0.48	NI	NI
C1_03270W_A	C1_03270W_A	0.27	NI	NI
C1_03450C_A	C1_03450C_A	0.44	NI	NI
C1_04700C_A	C1_04700C_A	0.23	NI	NI
C1_04960C_A	C1_04960C_A	0.31	NI	NI
C1_05300C_A	PRD1	0.26	NI	NI
C1_05540C_A	C1_05540C_A	0.37	NI	0.36
C1_05560W_A	RIB4	0.48	NI	NI
C1_06520C_A	BPH1	0.39	NI	NI
C1_06660W_A	C1_06660W_A	NI	0.43	1.30
C1_06810W_A	CAT1	NI	0.41	0.54
C1_06850W_A	PCL7	0.44	NI	NI
C1_07100C_A	C1_07100C_A	0.49	NI	NI
C1_07230W_A	PHO15	NI	0.41	0.75
C1_07350C_A	GPX3	0.44	NI	NI
C1_07940W_A	C1_07940W_A	0.38	NI	NI
C1_07980C_A	C1_07980C_A	0.25	0.50	0.70
C1_07990C_A	C1_07990C_A	NI	0.17	0.62
C1_08060W_A	OFR1	0.41	NI	NI
C1_08430W_A	CAF16	0.49	NI	0.46
C1_08830C_A	C1_08830C_A	0.24	NI	NI
C1_08840W_A	C1_08840W_A	0.49	NI	NI
C1_08880W_A	HEM14	0.43	NI	NI
C1_08940C_A	MSN4	0.49	NI	0.90
C1_08960W_A	C1_08960W_A	0.33	NI	NI
C1_08980C_A	ZWF1	0.37	NI	0.78
C1_09000W_A	C1_09000W_A	0.31	NI	NI
C1_09150W_A	AOX2	0.43	NI	NI
C1_09240C_A	C1_09240C_A	0.35	NI	NI
C1_09250W_A	CRP1	0.28	0.94	0.73
C1_09380W_A	RIM20	0.36	NI	NI
C1_09400C_A	FTH1	0.29	NI	NI
C1_09520C_A	C1_09520C_A	0.23	NI	NI
C1_09650W_A	C1_09650W_A	0.32	NI	NI
C1_10040W_A	ERO1	0.48	NI	1.05
C1_10180C_A	ECM21	0.41	NI	NI
C1_10510W_A	C1_10510W_A	0.45	NI	NI
C1_10740C_A	ASR1	0.13	0.46	NI
C1_10820C_A	C1_10820C_A	0.45	NI	NI
C1_11180C_A	C1_11180C_A	0.48	NI	NI
C1_11200W_A	C1_11200W_A	NI	0.34	NI
C1_11270W_A	C1_11270W_A	NI	0.50	NI
C1_11470C_A	DPP1	0.26	NI	NI
C1_11700C_A	MRF1	0.41	NI	0.94

C1_12060C_A	C1_12060C_A	0.47	NI	2.25
C1_12070C_A	C1_12070C_A	0.27	NI	1.60
C1_12240C_A	C1_12240C_A	0.38	NI	NI
C1_12730W_A	RVS162	0.25	NI	NI
C1_12850W_A	BLP1	0.08	NI	NI
C1_12970C_A	SEC2	0.41	NI	0.41
C1_13160W_A	PSA2	0.47	NI	NI
C1_13270W_A	C1_13270W_A	0.29	NI	NI
C1_13470W_A	KNS1	0.12	NI	NI
C1_13480W_A	HSP70	0.16	0.67	NI
C1_14030W_A	C1_14030W_A	0.34	NI	NI
C1_14040W_A	C1_14040W_A	0.42	NI	NI
C1_14180W_A	C1_14180W_A	0.26	NI	NI
C1_14190C_A	C1_14190C_A	0.43	NI	NI
C2_00530W_A	C2_00530W_A	0.05	NI	NI
C2_00540W_A	C2_00540W_A	0.18	NI	0.77
C2_00580C_A	SMF3	NI	NI	0.43
C2_00760C_A	C2_00760C_A	0.24	NI	NI
C2_01010W_A	HGT8	NI	0.48	1.01
C2_01270W_A	CHA1	0.47	NI	NI
C2_02010C_A	CHT4	0.40	NI	NI
C2_02850W_A	UGA6	0.32	NI	NI
C2_02860W_A	SUR2	NI	NI	0.49
C2_02970C_A	ALD5	NI	0.35	NI
C2_03020C_A	C2_03020C_A	NI	0.29	NI
C2_03030W_A	LIG4	0.40	NI	NI
C2_03170W_A	C2_03170W_A	0.38	NI	NI
C2_03260W_A	C2_03260W_A	0.29	NI	NI
C2_04010C_A	HSP21	0.17	NI	0.51
C2_06550W_A	C2_06550W_A	0.28	NI	NI
C2_06600W_A	C2_06600W_A	0.30	NI	NI
C2_06720W_A	GRE2	0.19	NI	2.24
C2_06890C_A	C2_06890C_A	0.28	0.57	NI
C2_06940C_A	ARE2	0.45	NI	NI
C2_07070W_A	C2_07070W_A	0.39	NI	1.85
C2_07140W_A	C2_07140W_A	0.42	NI	NI
C2_07630C_A	C2_07630C_A	0.33	0.49	NI
C2_08120W_A	MAF1	0.44	NI	NI
C2_08130W_A	ARA1	0.25	NI	NI
C2_08260W_A	C2_08260W_A	0.10	NI	NI
C2_08290C_A	UCF1	0.34	NI	1.92
C2_08300C_A	C2_08300C_A	0.22	NI	NI
C2_08390W_A	C2_08390W_A	0.29	NI	0.57
C2_08420W_A	C2_08420W_A	0.37	NI	NI
C2_08860W_A	C2_08860W_A	0.36	NI	0.53
C2_09480W_A	C2_09480W_A	0.36	NI	NI
C2_09590C_A	C2_09590C_A	0.26	NI	NI
C2_09860C_A	C2_09860C_A	0.29	NI	NI
C2_10450W_A	NCE4	0.44	NI	NI
C3_00220W_A	HGT19	NI	0.45	NI
C3_00230C_A	C3_00230C_A	NI	0.31	NI
C3_00320W_A	RHR2	NI	0.43	1.48
C3_00600W_A	IFF11	0.35	NI	NI
C3_00930W_A	ATO2	NI	NI	0.39
C3_01280W_A	C3_01280W_A	0.40	NI	NI
C3_01540W_A	C3_01540W_A	0.27	NI	NI
C3_01820W_A	C3_01820W_A	0.46	NI	NI
C3_01930W_A	PXP2	0.20	NI	NI
C3_02140C_A	C3_02140C_A	0.32	0.38	NI
C3_02480C_A	CCP1	0.40	NI	NI
C3_02610C_A	GLX3	0.30	0.56	0.32
C3_03720W_A	GTT11	0.25	NI	1.41
C3_04150W_A	C3_04150W_A	0.34	NI	NI
C3_04550C_A	CMK1	0.40	NI	0.76

C3_04650W_A	C3_04650W_A	0.30	NI	NI
C3_04970C_A	C3_04970C_A	0.41	NI	NI
C3_05080W_A	C3_05080W_A	0.27	NI	NI
C3_05090C_A	C3_05090C_A	0.31	NI	NI
C3_05360C_A	C3_05360C_A	0.26	NI	NI
C3_06180C_A	TSA1	0.13	NI	1.85
C3_06270C_A	C3_06270C_A	0.40	NI	0.93
C3_06330W_A	TSA1B	0.08	NI	NI
C3_06490W_A	C3_06490W_A	0.42	NI	0.63
C3_06700C_A	C3_06700C_A	NI	NI	0.48
C3_06920W_A	C3_06920W_A	0.32	NI	NI
C3_07270C_A	UBI4	0.50	NI	NI
C3_07330W_A	C3_07330W_A	0.50	NI	NI
C4_00120W_A	PGA7	0.16	NI	NI
C4_00150C_A	PEX5	0.31	NI	NI
C4_00580W_A	C4_00580W_A	0.45	NI	NI
C4_00950C_A	C4_00950C_A	0.17	NI	NI
C4_01100C_A	AGP2	NI	0.35	0.55
C4_01620C_A	C4_01620C_A	0.19	NI	NI
C4_01690C_A	HRT2	0.22	NI	NI
C4_01860C_A	C4_01860C_A	0.17	NI	NI
C4_01970W_A	C4_01970W_A	0.26	NI	NI
C4_02100C_A	GPI14	NI	NI	0.43
C4_02330C_A	C4_02330C_A	0.04	NI	NI
C4_02360W_A	AMS1	0.20	NI	NI
C4_02990C_A	GST2	0.44	NI	1.52
C4_03100W_A	RBT7	0.49	NI	NI
C4_03200C_A	C4_03200C_A	0.48	NI	1.00
C4_03370C_A	C4_03370C_A	0.15	NI	NI
C4_03430W_A	MOH1	0.12	NI	0.38
C4_03600C_A	C4_03600C_A	0.17	NI	NI
C4_03890W_A	PTP2	0.28	NI	NI
C4_03960W_A	C4_03960W_A	0.38	NI	NI
C4_04710W_A	GYP1	0.35	NI	NI
C4_05140C_A	GDB1	0.35	NI	NI
C4_05390W_A	C4_05390W_A	0.26	NI	NI
C4_06390W_A	SOU1	NI	0.28	NI
C4_06780C_A	OYE32	0.36	NI	1.00
C4_06890W_A	ARR3	0.12	NI	1.39
C4_06900W_A	GST1	0.09	NI	NI
C5_00710W_A	IFF8	0.33	NI	NI
C5_00750C_A	C5_00750C_A	0.43	NI	NI
C5_00880C_A	GIT3	0.46	NI	NI
C5_00930C_A	TFS1	0.35	NI	NI
C5_01920C_A	C5_01920C_A	0.45	NI	NI
C5_02080C_A	HSP12	NI	0.48	NI
C5_02380W_A	C5_02380W_A	NI	0.28	NI
C5_02630C_A	MNN1	0.13	NI	NI
C5_02690W_A	C5_02690W_A	NI	NI	0.44
C5_02860C_A	GRP2	0.23	NI	1.44
C5_02930C_A	GRE3	0.41	NI	NI
C5_03240W_A	BUB3	0.34	NI	0.69
C5_03490C_A	C5_03490C_A	0.18	NI	NI
C5_04050W_A	C5_04050W_A	0.44	NI	NI
C5_04220W_A	MRV5	0.09	NI	NI
C5_04360C_A	C5_04360C_A	0.25	NI	NI
C5_04370C_A	PGA37	0.26	NI	NI
C5_04870W_A	C5_04870W_A	0.28	NI	NI
C5_05430W_A	PEX4	0.36	NI	NI
C5_05450C_A	MUM2	0.22	NI	NI
C5_05480W_A	DES1	0.31	NI	NI
C6_00220C_A	C6_00220C_A	0.35	NI	NI
C6_01300W_A	C6_01300W_A	0.42	NI	0.45
C6_01420C_A	C6_01420C_A	0.48	NI	1.25

C6_01510W_A	OYE23	0.20	NI	1.12
C6_01750C_A	C6_01750C_A	0.49	NI	NI
C6_01990W_A	PLB1	0.21	NI	NI
C6_02010C_A	GPD2	NI	0.34	0.89
C6_02030C_A	C6_02030C_A	0.24	NI	NI
C6_02420W_A	C6_02420W_A	0.12	NI	NI
C6_02480W_A	C6_02480W_A	0.32	0.31	1.24
C6_02500C_A	GCV1	0.50	NI	NI
C6_02950C_A	C6_02950C_A	0.14	NI	NI
C6_03270C_A	AYR2	0.28	NI	NI
C6_03790C_A	HGT10	NI	0.20	NI
C7_00350C_A	C7_00350C_A	0.37	NI	1.42
C7_00760C_A	C7_00760C_A	0.34	NI	1.00
C7_00770W_A	C7_00770W_A	0.19	NI	6.67
C7_01230C_A	C7_01230C_A	0.43	NI	1.02
C7_01940C_A	C7_01940C_A	NI	0.37	0.61
C7_02220C_A	C7_02220C_A	0.42	NI	NI
C7_02330W_A	YCF1	0.41	NI	NI
C7_02450W_A	C7_02450W_A	0.44	NI	NI
C7_02520W_A	C7_02520W_A	0.32	NI	NI
C7_02910W_A	ENA21	NI	0.40	NI
C7_03240W_A	C7_03240W_A	0.44	NI	NI
C7_03450C_A	HSM3	0.29	NI	0.62
C7_03520W_A	PNC1	0.48	NI	NI
C7_03580C_A	C7_03580C_A	0.28	NI	0.81
C7_03680W_A	C7_03680W_A	0.43	NI	NI
C7_03780C_A	C7_03780C_A	0.07	NI	NI
C7_03860W_A	C7_03860W_A	0.16	NI	NI
CM_00190C	tC(GCA)3mt	0.14	NI	NI
CR_01200W_A	OYE22	0.32	NI	1.83
CR_02180W_A	PHO113	0.28	NI	NI
CR_02240C_A	OPT2	0.31	NI	0.88
CR_02650C_A	DRE2	0.49	NI	2.66
CR_02960W_A	CR_02960W_A	0.41	NI	NI
CR_03220C_A	CR_03220C_A	0.46	NI	NI
CR_03280W_A	IFR2	NI	0.45	1.17
CR_04060C_A	DAP1	0.40	NI	0.42
CR_04350C_A	CR_04350C_A	0.43	NI	NI
CR_04960C_A	CRG1	NI	NI	0.45
CR_05440W_A	CR_05440W_A	0.39	NI	NI
CR_05740C_A	PTC4	0.49	NI	NI
CR_06730W_A	APG7	0.31	NI	NI
CR_07150W_A	GLK1	0.24	NI	0.87
CR_07160C_A	CR_07160C_A	0.38	NI	NI
CR_07480W_A	CR_07480W_A	0.27	NI	0.95
CR_07490C_A	GLK4	0.27	NI	NI
CR_07790C_A	YHB1	0.43	NI	NI
CR_08250C_A	HSP104	0.37	NI	NI
CR_08310C_A	CR_08310C_A	0.17	NI	NI
CR_08700C_A	ARF1	0.36	NI	NI
CR_08890C_A	ASR2	NI	0.43	NI
CR_08990C_A	CR_08990C_A	NI	0.49	NI
CR_09140C_A	CR_09140C_A	NI	0.41	NI
CR_09270C_A	CBP1	0.37	0.91	0.43
CR_09680C_A	RTA4	0.45	NI	NI
CR_10200W_A	CR_10200W_A	0.05	NI	NI
CR_10350C_A	TRX1	0.42	NI	1.95
