# Supplementary material for: Redox Regulation, Rather than Stress-Induced Phosphorylation, of a Hog1 Mitogen-Activated Protein Kinase Modulates Its Nitrosative-Stress-Specific Outputs
Source: mBio. 2018 Mar 27;9(2):e02229-17. doi: 10.1128/mBio.02229-17 (PMC5874921; doi:10.1128/mBio.02229-17)
Supplement: TABLE S5 [file mbo002183795st5.pdf]

## Table S5. Oxidative and osmotic stress responses of Hog1-dependent genes that retain inducibility in response to nitrosative stress

Hog1-dependent NS genes were defined as displaying a >2-fold decrease in their nitrosative stress induction following Hog1 inactivation (i.e. fold-induction in *hog1* cells/fold-induction in wild type cells)

We selected Hog1-dependent NS genes that retain at least partial induction (i.e. >2-fold induction) in *hog1* cells in response to nitrosative stress. We then examined their induction in response to oxidative stress (XS) or osmotic stress (OS) using the data from Enjalbert *et al.* (2006).

Yellow = induced in response to XS or OS

**ND** = no data for this gene

Enjalbert *et al.* (2006) Molec. Biol. Cell 17, 1018-1032

| GENE | | **WT+NS/ WT** | ***hog1* +NS/ *hog1*** | **(*hog1* +NS/*hog1* )/ (WT+NS/WT)** | **XS** | **OS** |
|---|---|---|---|---|---|---|
| | | | (this study) | | (Enjalbert et al., 2006) | |
| C1_01220C_A | C1_01220C_A | 9.2 | 3.5 | 0.38 | 1.0 | 1.0 |
| C1_05540C_A | C1_05540C_A | 5.8 | 2.1 | 0.37 | 5.3 | 1.4 |
| C1_06520C_A | BPH1 | 5.3 | 2.1 | 0.39 | 1.6 | 1.4 |
| C1_08430W_A | CAF16 | 6.8 | 3.3 | 0.49 | 4.4 | 1.3 |
| C1_08940C_A | MSN4 | 4.7 | 2.3 | 0.49 | 2.7 | 1.6 |
| C1_09150W_A | AOX2 | 16.4 | 7.1 | 0.43 | 1.2 | 1.0 |
| C1_10740C_A | ASR1 | 7.7 | 2.7 | 0.35 | 1.1 | 4.8 |
| C1_11700C_A | MRF1 | 16.3 | 6.7 | 0.41 | 10.3 | 1.6 |
| C1_13480W_A | HSP70 | 17.8 | 2.9 | 0.16 | 0.9 | 3.2 |
| C2_00530W_A | C2_00530W_A | 20.3 | 2.6 | 0.13 | ND | ND |
| C2_00760C_A | C2_00760C_A | 54.0 | 13.1 | 0.24 | ND | ND |
| C2_02850W_A | UGA6 | 11.6 | 3.7 | 0.32 | ND | ND |
| C2_04010C_A | HSP21 | 5.9 | 2.3 | 0.39 | 3.2 | 1.0 |
| C2_06940C_A | ARE2 | 7.0 | 3.1 | 0.45 | 1.7 | 1.9 |
| C2_07070W_A | C2_07070W_A | 12.7 | 4.9 | 0.39 | 4.8 | 1.1 |
| C2_07630C_A | C2_07630C_A | 8.7 | 2.8 | 0.33 | 1.5 | 2.1 |
| C2_08390W_A | C2_08390W_A | 20.7 | 5.9 | 0.29 | 2.9 | 1.6 |
| C2_08420W_A | C2_08420W_A | 7.0 | 2.6 | 0.37 | 1.0 | 1.1 |
| C3_00600W_A | IFF11 | 10.3 | 3.6 | 0.35 | 1.1 | 1.0 |
| C3_02480C_A | CCP1 | 7.4 | 2.9 | 0.40 | ND | ND |
| C3_06180C_A | TSA1 | 8.0 | 3.0 | 0.38 | 3.1 | 1.0 |
| C3_06490W_A | C3_06490W_A | 9.4 | 4.0 | 0.42 | 3.1 | 1.6 |
| C3_07330W_A | C3_07330W_A | 4.2 | 2.1 | 0.50 | 1.2 | 1.1 |
| C4_02360W_A | AMS1 | 17.3 | 3.4 | 0.20 | 1.7 | 1.3 |
| C4_02990C_A | GST2 | 13.9 | 6.1 | 0.44 | 5.3 | 1.0 |
| C4_03100W_A | RBT7 | 8.2 | 4.0 | 0.49 | 1.0 | 1.0 |
| C4_03960W_A | C4_03960W_A | 7.3 | 2.8 | 0.38 | ND | ND |
| C4_06780C_A | OYE32 | 28.5 | 10.4 | 0.36 | 8.7 | 1.2 |
| C4_06900W_A | GST1 | 105.0 | 9.1 | 0.09 | 1.9 | 1.0 |
| C5_03490C_A | C5_03490C_A | 5.5 | 2.0 | 0.36 | 1.4 | 1.5 |
| C6_01510W_A | OYE23 | 27.4 | 5.4 | 0.20 | 3.7 | 1.1 |
| C6_01990W_A | PLB1 | 32.5 | 6.8 | 0.21 | 0.4 | 0.3 |
| C7_00760C_A | C7_00760C_A | 6.3 | 2.2 | 0.34 | 2.0 | 1.0 |
| C7_00770W_A | C7_00770W_A | 22.5 | 4.3 | 0.19 | 2.2 | 1.0 |
| C7_03580C_A | C7_03580C_A | 10.5 | 2.9 | 0.28 | 2.2 | 1.0 |
| CR_04060C_A | DAP1 | 6.1 | 2.5 | 0.40 | 4.0 | 1.8 |
| CR_07150W_A | GLK1 | 11.7 | 2.8 | 0.24 | 2.0 | 1.6 |
| CR_07160C_A | CR_07160C_A | 5.8 | 2.2 | 0.38 | ND | ND |
| CR_07480W_A | CR_07480W_A | 8.6 | 2.3 | 0.27 | 2.9 | 1.5 |
| CR_07490C_A | GLK4 | 10.9 | 2.9 | 0.27 | ND | ND |
| CR_07790C_A | YHB1 | 18.5 | 7.9 | 0.43 | 1.8 | 1.1 |
| CR_10200W_A | CR_10200W_A | 19.5 | 7.3 | 0.37 | 1.0 | 1.0 |
| CR_10350C_A | TRX1 | 5.4 | 2.3 | 0.42 | 3.1 | 0.9 |
